# Engineered multiple translation initiation sites: a novel tool to enhance protein production in *Bacillus licheniformis* and other industrially relevant bacteria

**DOI:** 10.1093/nar/gkac1039

**Published:** 2022-11-16

**Authors:** Manyu Zhang, Jing Song, Jun Xiao, Jingjie Jin, Christopher T Nomura, Shouwen Chen, Qin Wang

**Affiliations:** State Key Laboratory of Biocatalysis and Enzyme Engineering, Environmental Microbial Technology Center of Hubei Province, College of Life Science, Hubei University, Wuhan 430062, China; State Key Laboratory of Biocatalysis and Enzyme Engineering, Environmental Microbial Technology Center of Hubei Province, College of Life Science, Hubei University, Wuhan 430062, China; State Key Laboratory of Biocatalysis and Enzyme Engineering, Environmental Microbial Technology Center of Hubei Province, College of Life Science, Hubei University, Wuhan 430062, China; Key Laboratory of Functional Protein Research of Guangdong Higher Education Institutes, Institute of Life and Health Engineering, College of Life Science and Technology, Jinan University, Guangzhou 510632, China; Department of Biological Sciences, University of Idaho, 875 Perimeter Drive, Moscow, ID 83844, USA; State Key Laboratory of Biocatalysis and Enzyme Engineering, Environmental Microbial Technology Center of Hubei Province, College of Life Science, Hubei University, Wuhan 430062, China; State Key Laboratory of Biocatalysis and Enzyme Engineering, Environmental Microbial Technology Center of Hubei Province, College of Life Science, Hubei University, Wuhan 430062, China

## Abstract

Gram-positive bacteria are a nascent platform for synthetic biology and metabolic engineering that can provide new opportunities for the production of biomolecules. However, the lack of standardized methods and genetic parts is a major obstacle towards attaining the acceptance and widespread use of Gram-positive bacterial chassis for industrial bioproduction. In this study, we have engineered a novel mRNA leader sequence containing more than one ribosomal binding site (RBS) which could initiate translation from multiple sites, vastly enhancing the translation efficiency of the Gram-positive industrial strain *Bacillus licheniformis*. This is the first report elucidating the impact of more than one RBS to initiate translation and enhance protein output in *B. licheniformis*. We also explored the application of more than one RBS for both intracellular and extracellular protein production in *B. licheniformis* to demonstrate its efficiency, consistency and potential for biotechnological applications. Moreover, we applied these concepts for use in other industrially relevant Gram-positive bacteria, such as *Bacillus subtilis* and *Corynebacterium glutamicum*. In all, a highly efficient and robust broad-host expression element has been designed to strengthen and fine-tune the protein outputs for the use of bioproduction in microbial cell factories.

## INTRODUCTION

The use of microorganisms for the industrial production of a number of platform chemicals is commonplace in modern society. In particular, the production of industrially relevant proteins at large scale is both challenging and important for economic viability. A group of generally regarded as safe (GRAS) Gram-positive bacteria including *Bacillus subtilis*, *Bacillus licheniformis* and *Corynebacterium glutamicum* are particularly favored for the production of biomolecules in the food and medical industries because of their non-toxicity (food grade) and the ease with which high cell densities and high productivity can be achieved. A variety of synthetic biology tools, such as constitutive and inducible promoters, ribosome-binding sites (RBSs) and mRNA 5′ stem–loops, have been created and evaluated in these strains (1–5), and several expression systems have been commercially available through the company MoBiTec (mobitec.com/products/vector-systems/). However, unlike the model bacterium *Escherichia coli*, a robust and highly efficient expression system has not been developed for use with these industrial microorganisms.

Translation efficiency (TE) depends on several factors, including the rate of translation initiation, the speed of translation elongation, the ribosomal loading capacity as well as the stability of mRNA and proteins (6–8). Translation initiation is often regarded as the rate-limiting phase and a major checkpoint for regulating the level and fidelity of protein synthesis (9,10). The free 3′ end of the 16S rRNA plays a pivotal role in translation initiation by base-pairing with the complementary ‘Shine–Dalgarno’ (SD) sequence located with an appropriate spacing upstream of the start codon on the mRNA (11,12).

To improve protein production in industrially relevant GRAS bacteria, we previously constructed a hairpin structure located immediately upstream of the start codon from the commonly used *B. subtilis* promoter P43, with a single-strand SD sequence present within the 10 nt loop of the hairpin for better ribosomal recruitment. This unique secondary structure effectively promoted protein output in *B. licheniformis*, and the expression levels of the target proteins increased 50-fold (13). In this work, we built upon those previous findings to place a multi-hairpin structure in tandem 5′ to the gene encoding green fluorescent protein (GFP), and protein translation was enhanced in parallel with the increased number of the hairpins. Since each hairpin structure contains an SD sequence and a start codon, theoretically they all could be used as RBSs to initiate translation. Alternative translation initiation sites (TISs) have been discovered in several eukaryotic cells, including yeast, mouse and human cells (14–16). Translation of the majority of bacterial genes is initiated from a unique TIS; however, there have been a few serendipitously discovered cases where an alternative TIS can direct translation (17–19). Recently, 124 potential alternative TISs were identified genome-wide in *E. coli* using Ribo-seq analysis, suggesting that initiation at alternative start sites is widespread in bacteria (20). Furthermore, the native leader sequences encoding mannanase A and cellulase A in *Streptomyces lividans* contain two potential RBSs that could be used to enhance the production of xylanase A (21). Commercially available protein expression systems for *Bacillus megaterium* through the company MoBiTec contain two RBSs for simultaneous dual expression (mobitec.com/products/vector-systems/). However, to the best of our knowledge, our study is the first to explore the impact of more than one RBS within mRNA leader sequences and their effect on the protein output in bacteria. The results of this study point to a novel broad-host expression tool that could be used to strengthen and fine-tune the protein outputs in several industrially relevant bacteria for synthetic biology and metabolic engineering uses.

## MATERIALS AND METHODS

### Strains, plasmids and growth conditions

Strains and plasmids used in this study are listed in Supplementary Tables S1 and S2. *Escherichia coli* DH5α was used for all cloning steps. *Bacillus licheniformis* DW2 and *B. subtilis* 168 were used as the expression hosts harboring the expression vector pHY300PLK. For GFP production, these strains were grown in Luria–Bertani (LB) broth for 24 h at 37°C, 230 rpm, with 20 mg/l tetracycline. *Corynebacterium glutamicum* 13032 was used as the protein expression host harboring the plasmid pEC-XK99E; it was grown on LB agar plates or in LB broth for 24 h at 30°C, 180 rpm, with 10 mg/l kanamycin when necessary. *Escherichia coli* BL21(DE3) was used as the protein expression host carrying the plasmid pET28a, which were grown in LB broth for 24 h at 37°C, 230 rpm, with 25mg/l kanamycin.

The fermentation medium for keratinase production consisted of 2% glucose,1.5% yeast extract, 1% tryptone, 1% NaCl, 0.6% (NH4)_2_SO_4_, 1% corn steep liquor, 0.3% K_2_HPO_4_ and 1% soybean meal with a pH of 7.2 before sterilizing. The fermentation medium for arginase consisted of 2% glucose, 50 mM Tris, 0.6 mM KH_2_PO_4_, 2 mM CaCl_2_, 1 μM FeSO_4_·7H_2_O, 10 μM MnSO_4_·4H_2_O, 4.5 mM glutamic acid, 780 μM tryptophan, 860 μM lysine, 15 mM (NH_4_)_2_SO_4_, 8 mM MgSO_4_·7H_2_O, 27 mM KCl, 7 mM Na_3_C_6_H_5_O_7_·2H_2_O with a pH of 7.5 before sterilizing. The fermentation medium for hydroxytyrosol consisted of 10% glucose, 48 mM Na_2_HPO_4_·12H_2_O, 22 mM KH_2_PO_4_, 19 mM NH_4_Cl, 12.5 mM NaCl, 1 mM MgSO_4_·7H_2_O, 4 mM Na_3_C_6_H_5_O_7_, 3 mM yeast extract, 100 μM CaCl_2_·2H_2_O, 50 μM FeCl_3_·6H_2_O, 12.5 μM ZnCl_2_, 5 μM MnCl_2_·4H_2_O, 2.5 μM CoCl_2_·6H_2_O, 2 μM Na_2_MoO_4_·7H_2_O and 2.5 μM CuCl_2_·2H_2_O, and 2 g/l tyrosol was added at the mid-log phase of the fermentation with a pH of 7.0. The cells were grown for 48 h at 37°C, 230 rpm. All the fermentation experiments were repeated a minimum of three times.

### Construction of overexpression strains

In the process of constructing the expression vector of the multiple RBSs, the expression plasmid with an optimized 5′-untranslated region (UTR) and codon-optimized GFP from a previous study (13) was used as a template. The forward primer RBS-F contains the terminal 18 nt of the 5′-UTR and the first 18 nt of the GFP gene; reverse primer RBS-R is a two-copy 5′-UTR reverse sequence, and the 3′-end of the reverse primer was varied according to experimental requirements. The designed primers are listed in Supplementary Table S3. Since the RBS-R primer is a sequence with duplicate copies, it can be bridged end to end and extended continuously, resulting in a linear plasmid with multicopy repeats (Supplementary Figure S1). The linear vector obtained by polymerase chain reaction (PCR) was recovered, purified and circularized *in vitro* using the recombinase Exnase II (Vazyme Biotech Co., Ltd, Nanjing, China) according to the manufacturer's instruction. The circularized vector was then transformed into *E. coli* DH5α. The primer RBS-YF/YR was designed to screen transformants, and the number of tandem sequences was preliminarily judged based on the length of the colony PCR products. Plasmids were extracted from the positive transformants and DNA sequencing was performed to verify the tandem sequence expression vector. The arginase gene *rocF* (GeneBank accession no. CP005965.1, signal peptide replaced with PhoD), 4-hydroxyphenylacetate 3-monooxygenase genes *hpaBC* (GeneBank accession no. CP053602.1) and the keratinase gene from *Bacillus amyloliquefaciens* were independently cloned into the expression vector pHY300PLK using the recombinase Exnase II.

The expression plasmids were independently introduced into *B. licheniformis* DW2, *B. subtilis* 168 and *C. glutamicum* 13032 by electroporation. For the transformation of *B. licheniformis* and *B. subtilis*, the cells were grown in the growth medium (LB containing 0.5 M sorbitol) to an OD_600_ of 0.8–0.9. The cells were cooled on ice for 15 min and harvested by centrifugation at 4°C and 5500 *g* for 7 min. The harvested cells were washed in ice-cold wash medium (0.5 M sorbitol, 0.5 M mannitol and 12.6% glycerol) four times, and resuspended in 500 μl of wash medium. Transformation was carried out by electroporation with a 2.4 kV, 200 Ω, 25 μF electric pulse in a 0.2 cm cuvette using a Gene Pulser (Bio-Rad Laboratories, Hercules, CA, USA). A 1 ml aliquot of recovery medium (LB containing 0.5 M sorbitol and 0.38 M mannitol) was added, and incubated at 37°C, 110 rpm for 3 h. For the transformation of *C. glutamicum*, the cells were grown in medium (5% yeast extract, 10% tryptone, 10% NaCl, 30% glycine, 0.1% Tween-80, 0.4% d-Ala, pH 6.5) with the addition of 1 ml of 10% isoniazid. After incubating for 3 h, ampicillin was added to the final concentration of 50 mg/ml. The cells were then grown at 30°C until the OD_600_ reached 0.5, and the culture was cooled on ice for 15 min. The cells were washed three times with 5 ml of 15% (v/v) glycerol, and resuspended in 500 μl of 10% glycerol. Transformation of *C. glutamicum* was carried out by electroporation with a 2.5 kV, 200 Ω, 25 μF electric pulse in a 0.2 cm cuvette using a Gene Pulser. After electroporation,a aliquot of 1 ml LBHIS medium (2.5% yeast extract, 5% tryptone, 5% NaCl,18.5% Brain Heart Infusion, 91% sorbitol) was added, heat-shocked at 46°C for 6 min, and then incubated at 30°C, 100 rpm for 2 h. Transformants were first selected for tetracycline or kanamycin resistance, and the positive colonies were validated by PCR with the primer pair pHY-F/R or PEC-XK99E-F/R, respectively.All sequences are presented in Supplementary Data.

### Construction of the protease-integrated strain

The TEV-P (codon-optimized protease from *Tobacco etch virus*) gene was cloned into the temperature-sensitive vector T2(2)-Ori. The upstream, downstream and the target gene were amplified by PCR using the primers TEVp-AF/AR, TEVp-BF/BR andTEVp-F/R, respectively. These three fragments were ligated via splicing overlap-extension PCR (SOE-PCR) and ligated with the vector using the ClonExpress One Step Cloning Kit (Vazyme Biotech Co., Ltd), according to the manufacturer's instructions. The resulting plasmid was then electro-transferred into *B. licheniformis* DW2, and the positive transformants were subjected to a double crossover process to integrate the TEV-P gene into the chromosome as described in the previous study (22). The TEV-P-integrated strain was further verified by DNA sequence, and named DW2::TEVp.

### Protein expression and purification

For GFP protein expressed in *E. coli*, cells were grown to an OD ∼0.6 at 37°C, induced with 1 mM isoprpopyl-β-d-thiogalactopyranoside (IPTG), and temperature was lowered to 28°C to allow expression overnight. For GFP expressed in *B. licheniformis*, cells were grown for 24 h at 37°C, 230 rpm. Harvested cells were resuspended in Lysis Buffer (50 mM NaH_2_PO_4_, 300 mM NaCl, 10 mM imidazole, pH 8.0) and lysed by sonication. The supernatant was applied into an Ni-NTA column loaded with Ni-NTA and SP-Sepharose. After loading the column with 50 ml of lysis buffer, the column was washed with the wash buffer (50 mM NaH_2_PO_4_, 300 mM NaCl, 20–50 mM imidazole, pH 8.0). GFP was purified over an elution buffer using a gradient of 100–500 mM imidazole.

### SDS–PAGE analysis for protein production

For GFP, the concentration of the purified protein was determined and it was mixed with the loading buffer. The samples were boiled for 15 min and then 5 μl of the redissolved protein was subjected to sodium dodecylsulfate–polyacrylamide gel electrophoresis (SDS–PAGE) which was performed using a 4% acrylamide stacking gel and 12% acrylamide resolving gel. For keratinase and arginase, the protein was precipitated with trichloroacetic acid (TCA) from the fermentation supernatant, and the obtained pellet was resuspended in 45 μl of the sample dilution buffer. The software ImageJ was used to quantify the intensity of protein bands on an SDS–PAGE gel.

### Analysis of fluorescence intensity

The relative fluorescence intensity of the GFP-expressing recombinants was measured by fluorometry. A relative fluorescence intensity and cell optical density were determined using a Multi-Mode Microplate Reader (SpectraMax iD3; Molecular Devices). An excitation wavelength of 480 nm and an emission wavelength of 520 nm were used to determine the fluorescence intensity of GFP. The fluorescence intensity of the strain harboring the plasmid pHY300PLK was used as the background fluorescence level for subtraction. The cell density was measured at a wavelength of 600 nm, and the relative fluorescent unit was normalized by dividing the subtracted fluorescent reading by the OD_600_. The cells were cultured for 24 h, washed twice in phosphate-buffered saline (PBS) and diluted 1:10 with PBS. All assays were performed in triplicate and the results are listed in Supplymentary Data.

### Enzymatic assays

The keratinase assay was done using 1% azocasein as the substrate according to the previous study (23), with modification. The crude enzyme was prepared by 50-fold dilution of the fermentation supernatant. Briefly, 1 ml of diluted enzyme was mixed with 1 ml of 1% azocasein dissolved in 30 mM Tris–HCl buffer (pH 8.0). This mixture was incubated at 40°C for 1 h and the reaction was stopped by the addition of 2 ml of 20% TCA. Then the tubes were centrifuged for 3 min at 13 000 *g* at 20°C and the absorbency was measured at 280 nm. One unit of keratinase activity was defined as an increase in absorbency of 0.01 at 280 nm.

For arginase activity, the 40% l-arginine (pH 9.0) and 2 ml fermentation broth mixtures were incubated at 37°C for 10 min and then moved to 100°C for 10 min to terminate the reaction. The l-ornithine content in the reaction mixture was detected using the HPLC-ELSD system (Agilent Technologies, Agilent Technologies 1260 Infinity ELSD, USA) equipped with an Ultimate ® Amino Acid Plus Column (Welch Technology, 4.6 × 300 mm; 5 μm). One unit of enzyme activity is defined as the amount of enzyme that produces 1 μmol of l-ornithine/min at 37°C.

After whole-cell catalysis, the concentrations of hydroxytyrosol in the fermentation supernatant were determined by a HPLC system (Shimadzu Technolgy LC-M20A, Japan) equipped with a Hypersil ODS2 (EliteHPLC, 4.6 × 250 mm; 5 μm), and detected with a UV detector at 224nm. All the experiments were repeated a minimum of three times and the results are listed in Supplymentary Data.

### Determination of mRNA half-life by northern blots

Ten percent of seed cultures were inoculated into LB medium and grown to a cell density of 2.5 at OD_600_ in the mid-exponential phase. Then rifampicin (200 μg/ml) was added to the cell culture. Incubation was continued for 2 min and 2 ml of culture samples were withdrawn at the time points 0, 5, 10, 15 and 20 min. Total RNA was extracted from *B. licheniformis* cells using the Trizol reagent (Invitrogen, Carlsbad, CA, USA). The stability of mRNAs was determined by northern blots using the DIG Northern Starter Kit (Roche, Indianapolis, IN, USA) following the manufacturer's instructions. GFP riboprobe was generated by standard PCR with primers GFP(blot)-F and GFP(blot)-R, encompassing 584 nt in the middle of the eGFP gene. The riboprobe for 16S rRNA was generated by standard PCR with primers 16S-F and 16S-R, encompassing 354 nt in the middle of the 16S rRNA gene. For northern blot analysis, 5 μg of total RNA was separated under denaturing conditions in a 1% (w/v) agarose MOPS/formaldehyde gel. The half-life of mRNA was determined by semi-logarithmic graph. The slope of the best-fitting line determines the value of *k*_decay_. The half-life value is derived from the equation *T*_1/2_ = ln(2)/*k*_decay_. All assays were performed in triplicate.

### Polysome fractionation

*Bacillus licheniformis* cells were grown in LB medium at 37°C, at 200 rpm in an orbital shaker to an OD_600_ of 2.5, and then chloramphenicol was added into the medium to a final concentration of 20 μg/ml, after which the culture was allowed to grow under the same conditions for an additional 10 min, and then harvested by centrifugation at 4°C, 3000 *g* for 5 min. The collected cell pellet was resuspended by RC buffer [50 mM HEPES, 1 M KOAc, 12 mM Mg(OAc)_2_, 2 mM β-mercaptoethanol, 20 ng/ml chloramphenicol, 10 μg/ml lysozyme, pH 7.4], and chilled on ice for 30 min. The resuspended cells were disrupted by a high pressure cell disrupter, and the cell residues were removed by centrifugation at 4°C, 8000 *g*. The supernatant was collected and treated with DNase I for 10 min to remove the genomic DNA. The lysate was carefully layered onto the top of a 10–42% sucrose gradient, followed by centrifugation using a Beckman Optima L-100 XP ultracentrifuge for 5 h at 4°C, 138 900 *g*. After centrifugation, the gradients were collected as 1.5 ml/min fractions with continuous monitoring of absorbance at 254 nm, and the polysome profile charts were plotted. The collected fractions of the sucrose gradient were subjected to ultrafiltration via centrifugation to remove sucrose and concentrate the samples.

### Quantitative reverse transcription–PCR (RT–qPCR) analysis

For determination of transcriptional levels of GFP with either one or six RBSs, the cells were grown in LB medium to a cell density of 2.5 at OD_600_ in the mid-exponential phase. Total RNA was extracted from *B. licheniformis* cells using the Trizol reagent. The trace contaminating DNAs in the samples were treated with RNase-free DNase I (NEB, Ipswich, MA, USA). The RNA concentrations were determined on a NanoDrop 2000 spectrophotometer (Thermo Scientific, Wilmington, DE, USA). First-strand cDNA was amplified from 0.5 μg of total RNA using the PrimeScript II 1st Strand cDNA Synthesis Kit (Takara, Japan) according to the manufacturer's instruction. Primers for RT–qPCR were designed using Primer 3 (https://bioinfo.ut.ee/primer3-0.4.0/) and are listed in Supplementary Table S2. The qRCRs using the iTaq™ Universal SYBR® Green Supermix (BIO-RAD, USA) were performed in a CFX Connect™ Real-Time PCR Detection System (BIO-RAD, USA) under the following conditions: 95°C for 2 min, then 40 cycles of 95°C (5 s) and 60°C (30 s), followed by a melting curve program. The adenylate kinase gene (*adk*) and 16S rRNA gene served as the reference genes.All reported GFP transcriptional levels are the average of three measurements and listed in Supplimentary Data.

For detection of the GFP level in each polysome fraction, RNA was extracted from each fraction using NucleoZOL reagent, and then reverse transcribed by a TIANGEN Quantscript RT Kit according to the manufacturer's instruction. The resulting cDNA was used for qPCR with primers designed using BIO-RAD iTaq™ Universal SYBR Green Supermix, and 16S rRNA was used as the reference gene. All reported GFP transcriptional levels are the average of three measurements and listed in Supplimentary Data.

## RESULTS

### Multiple RBSs generate multiple TISs to enhance protein output in *B. licheniformis*

**Figure 1.**
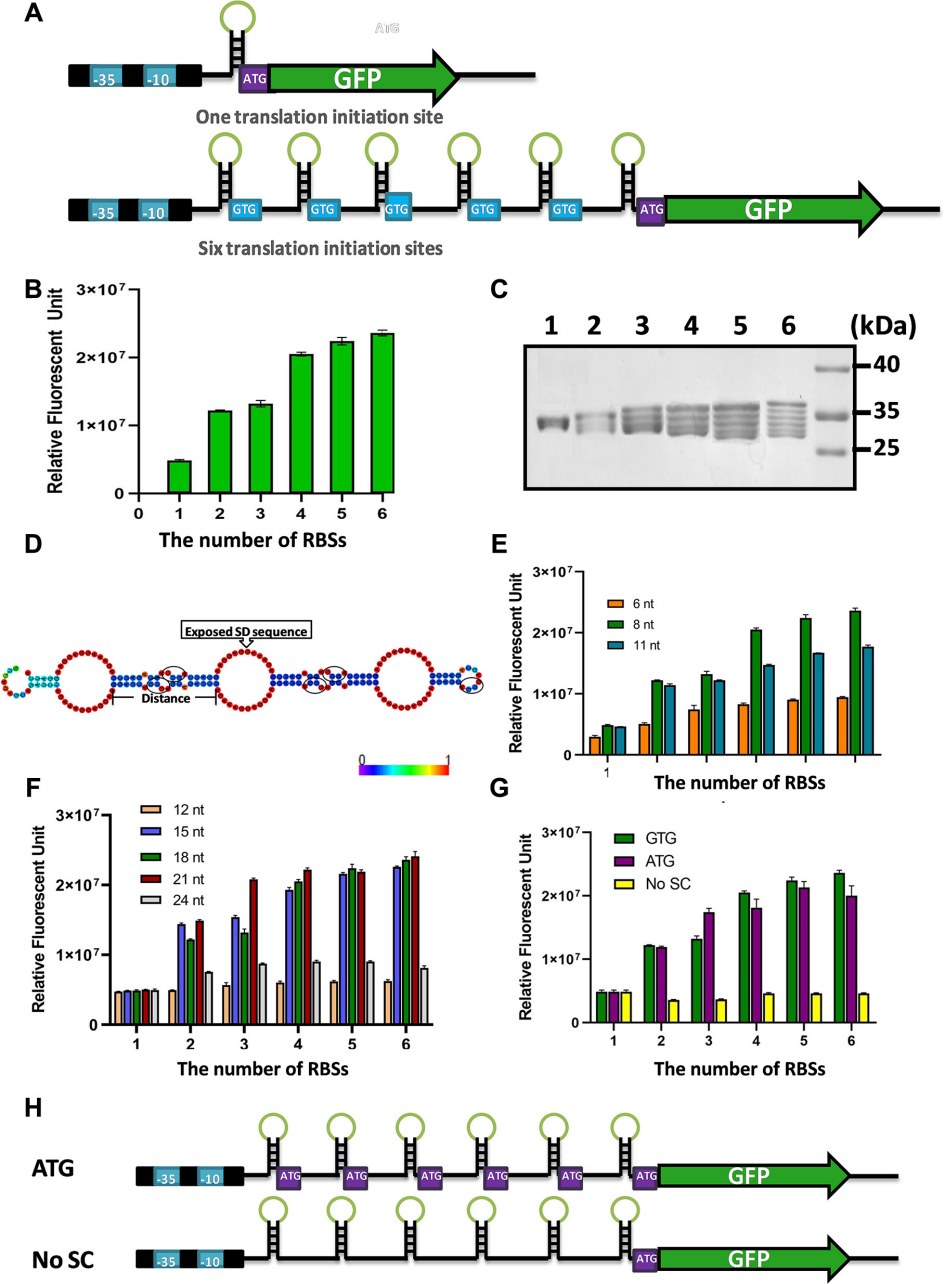
Protein expression was significantly enhanced as the number of RBSs increased in *B. licheniformis*. (**A**) Sequences with an RBS and start codon were located in tandem at the 5′-leader region of mRNAs. SD sequences located on the rings of the stems are highlighted in green. (**B**) The expression levels of GFP increased along with the increased number of RBSs. (**C**) Purified GFP proteins from *B. licheniformis* expressed with 1–6 RBSs. Lanes 1–6: the number of RBSs from one to six. (**D**) Predicted mRNA secondary structure of the sequence before the target gene. The secondary structure was predicted by RNAfold WebServer (rna.tbi.univie/cgi-bin/RNAWebSuite/RNAfold.cgi). Base-pair probabilities are represented by colors. Start codons are encircled in black. (**E**) The spacing between SD sequence and the start codon affected the protein output. (**F**) The distance between adjacent SD sequences impacted the protein output. (**G**) Start codons are essential for enhanced protein output. Green represents the multiple RBSs using GTG as the start codon, purple represents ATG as the start codon and yellow represents no start codon at the mRNA leading sequence but keeping the primary start codon ATG for the GFP gene. (**H**) Sequences of the six RBSs using ATG as the start codon and no start codon at the mRNA leading sequence. The experiments were performed in triplicate. The data are presented as the average numbers and the error bars are standard deviations.

In this study, we utilized GFP as an exemplar to examine the effects of more than one RBS engineered within an mRNA leader sequence on expression levels in the industrial GRAS strain, *B. licheniformis* (Figure 1A). We developed a one-step method for ligation of tandem repeat sequences (Supplementary Figure S1). Using this method, we constructed the expression plasmids carrying GFP genes with different numbers of RBSs within the mRNA leader region. Results indicated that the protein expression levels increased and were positively correlated to the number of RBSs. The fluorescent intensity improved dramatically when the RBS number increased within an mRNA leader sequence from one to four, with diminishing increases in fluorescent intensity for those mRNA leader sequences where the number of RBSs increased from four to six (Figure 1B). When the number of RBSs within an mRNA leader sequence reached six, the fluorescence intensity of GFP produced was 5-fold higher than that of an mRNA sequence with a single RBS (Figure 1B). The amount of GFP with six RBSs took up >50% of the total intracellular protein, which was the highest GFP production in *B. licheniformis* to the best of our knowledge (Supplementary Figure S2). However, the fluorescent intensity did not increase when the mRNA leader sequence was engineered to have more than six RBSs (Supplementary Figure S3). To determine the molecular weight of the expressed GFPs, the GFP genes were fused with sequences encoding a His-tag at the C-terminus, then purified, and analyzed by SDS–PAGE (Figure 1C). The results showed that the number of purified protein bands corresponded to the number of RBSs at the 5′-UTR, indicating that each RBS was used as a TIS. To investigate the causes of enhanced protein expression by more than one RBS, we examined the transcription levels and mRNA stability for the GFP genes with a single RBS and six RBSs by RT–qPCR and northern blots, respectively. There were no significant differences in the transcription levels and mRNA stabilities of the GFP genes between the two strains expressing ORFs with either a single RBS or six RBSs (Supplementary Figure S4), ruling out these factors as the causes of higher protein output. There were also no significant differences in cell growth between the strains with different RBSs, and the upward trend of GFP expression correlated with the increased number of RBSs was observed at different growth stages (Supplementary Figure S5). Therefore, the increased number of TISs was most probably responsible for the higher protein expression levels.

Based on the prediction of the RNAfold WebServer, the secondary structure of the six RBSs contained three cyclic tertiary structures composed of two exposed SD sequences, connected by double-stranded stems (Figure 1D). When we altered the secondary structure of two RBSs and sequestered one SD sequence (Supplementary Figure S6), the fluorescent intensity of GFP produced was similar to that of the GFP produced from the original mRNA structure (Supplementary Figure S5). This result suggested that the exposure of the SD sequences in the leader mRNA plays a minor role in TE. We also changed the DNA sequences of the six RBSs to make them non-repetitive. The results showed that the protein output dropped significantly when the primary structure for the N-terminus was changed significantly (Supplementary Figure S7). If only the DNA sequence was changed through codon substitution, while encoding a similar amino acid sequence, there was no significant difference in protein output compared with the original sequence. Therefore, the repetitive sequences within the mRNA leader region seemed to play little role in changing the TE, but the amino acid sequence at the N-terminus might have a large effect on protein output.

As expected, the decreased complementarity of the SD sequence to the ribosomes led to a significant decline of the protein output (Supplementary Figure S8). Likewise, the highest translational yield was achieved with engineered mRNA leader sequences with an 8 nt spacing (Figure 1E), which was in good agreement with previous observations that the optimal spacing for translation was 7–9 nt in both *B. subtilis* and *E. coli* (24). The distance between two SD sequences also had a profound influence on translational yield. We investigated distances ranging from 12 to 24 nt, and the results showed that only the distances from 15 to 21 nt could effectively boost protein output (Figure 1F). The necessity of the start codon was also investigated by substituting the original GUG start codon with the stronger AUG start codon and removing the start codon altogether at the mRNA leader sequence but keeping the original start codon of GFP. The replacement of GUG by AUG had little effect on protein expression, but the absence of start codon completely in the mRNA leader sequence abolished the positive effect of the multiple RBSs on translational yield (Figure 1G, H). Therefore, the start codon was a requisite element for multiple RBSs, and it also supported the hypothesis that multiple TISs in the leader region of the gene were the major determinant for the enhanced protein expression.

### Multiple RBSs promote translation efficiency via enhanced ribosome recruiting capacity

**Figure 2.**
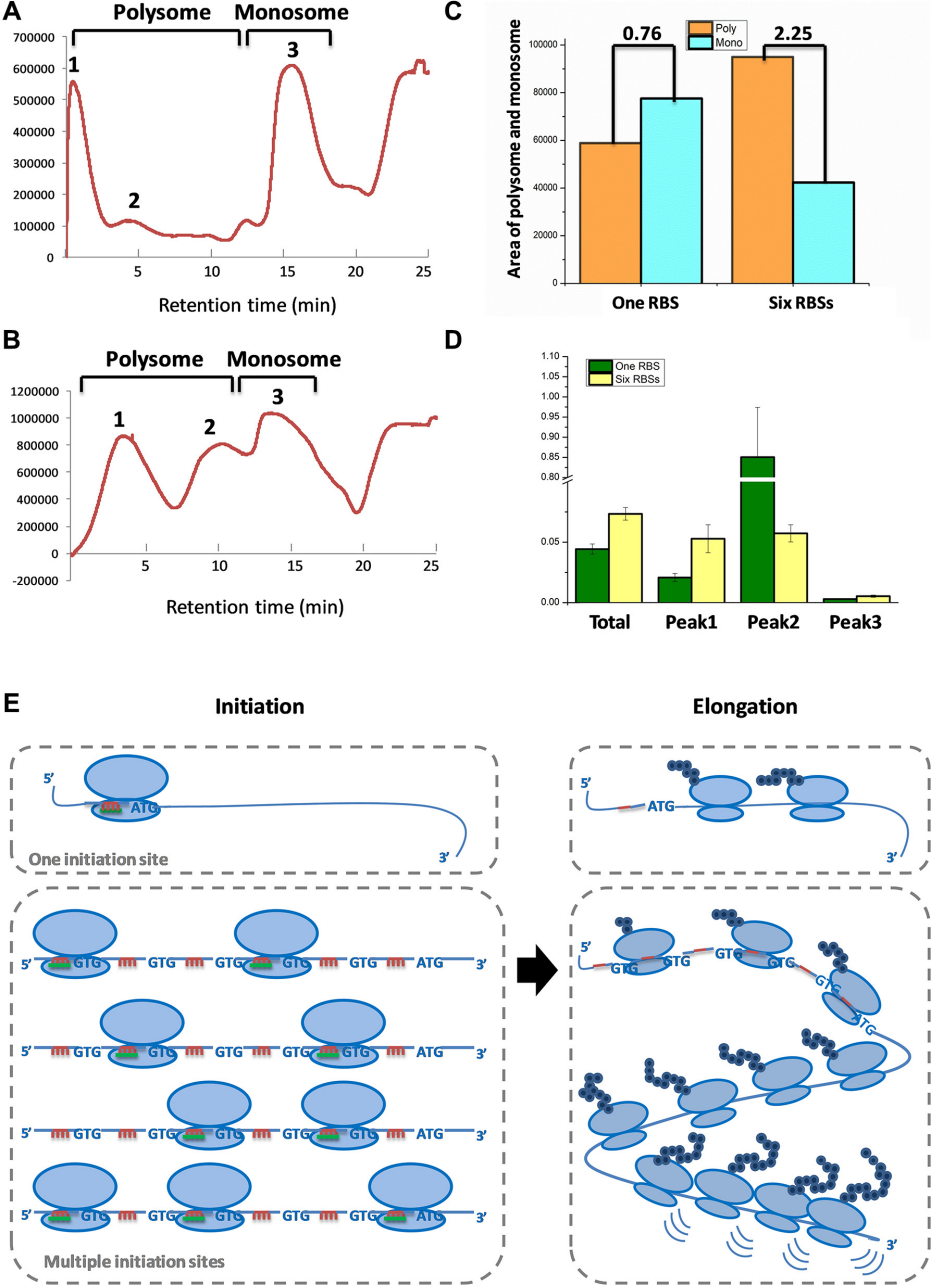
Polysome profiling experiments of *B. licheniformis* harboring the GFP gene with one or six RBSs. (**A**) Polysome gradient profiling of cells harboring the GFP gene with one RBS. (**B**) Polysome gradient profiling of cells harboring the GFP gene with six RBSs. Fractions from the bottom (42%) to the top (10%) of the gradient are shown from left to right. The polysomes and monosomes are indicated (Peak 1 and 2 are considered as the polysomes, and Peak 3 is considered as the monosomes). (**C**) The areas under the curve of polysomes and monosomes were measured, and the polysome:monosome ratio was calculated. (**D**) GFP mRNA presence in each fraction of the translated mRNAs was detected by RT–qPCR. (**E**) The model of how one RBS and six RBSs function in translation initiation and elongation. The red lines represent the SD sequences, and the green lines represent the 3′ end of the 30S ribosomal subunit, which are complementary to the SD sequences. The experiments were performed in triplicate. The data are presented as the average numbers and the error bars are standard deviations.

Polysome profiling is a powerful tool for the direct determination of TEs (25), providing valuable information about the translational status of specific mRNAs depending on the number of associated ribosomes. Generally, mRNAs associated with many ribosomes form large polysomes that are predicted to be robustly translated, whereas mRNAs associated with few or no ribosomes are expected to be translated poorly or remain untranslated. Therefore, we evaluated the translation status of cells expressing the GFP genes with either one or six RBSs using polysome profiling. The results distinctly showed the different ribosome distribution pattern between the cells harboring the GFP gene with one RBS versus cells expressing GFP with six RBSs (Figure 2A, B). The ratio of polysome to monosome increases from 0.76 to 2.25 when the numbers of RBSs for GFP were increased from one to six, indicating that the expression enhancement of the GFP gene caused by the multiple RBSs was so dramatic as to affect the TE at the whole-cell level (Figure 2C). Furthermore, the levels of GFP mRNA were determined by RT–qPCR in two polysome peaks and one monosome peak, using 16S rRNA as the reference gene (Figure 2D). The overall GFP mRNA level among the total translated mRNAs was significantly higher in the cells with six RBSs. The translated single RBS GFP mRNA was predominantly found in Peak 2, whereas the mRNA with the six RBS GFP transcripts was evenly distributed among two polysome peaks (Figure 2D). These results suggest that the GFP mRNAs with six RBSs had higher ribosome density than those with a single RBS, and corresponded to a higher protein synthesis rate. Furthermore, high ribosome density might cause ribosome collisions that would reduce the protein output, which could explain the limitations on GFP fluorescence when the number of RBSs exceeded six per transcript. The polysome fractionation analysis provides solid evidence that mRNA sequences with multiple RBSs distinctively change the translation pattern of the target gene and improve the TE significantly.

Combining the data of protein outputs and polysome profiling, we generated a model of how multiple RBSs function on translation initiation and elongation steps (Figure 2E). Compared with mRNA with a single RBS, more RBSs generate multiple initiation sites, resulting in higher translation initiation rates. Since translation is predominantly regulated at the initiation stage, the protein outputs increase gradually together with the increase of RBSs up to six copies. However, during elongation, more than six copies of RBSs generate higher ribosome density, which potentially could cause ribosome collisions at the end of the mRNAs analogous to a traffic jam (26). The ribosome queueing events could impede the rate of protein synthesis, causing the saturation of protein outputs, and stop growing when there are more than six RBS copies.

### Multiple RBSs were an effective tool for extracellular and intracellular protein production in *B. licheniformis*

**Figure 3.**
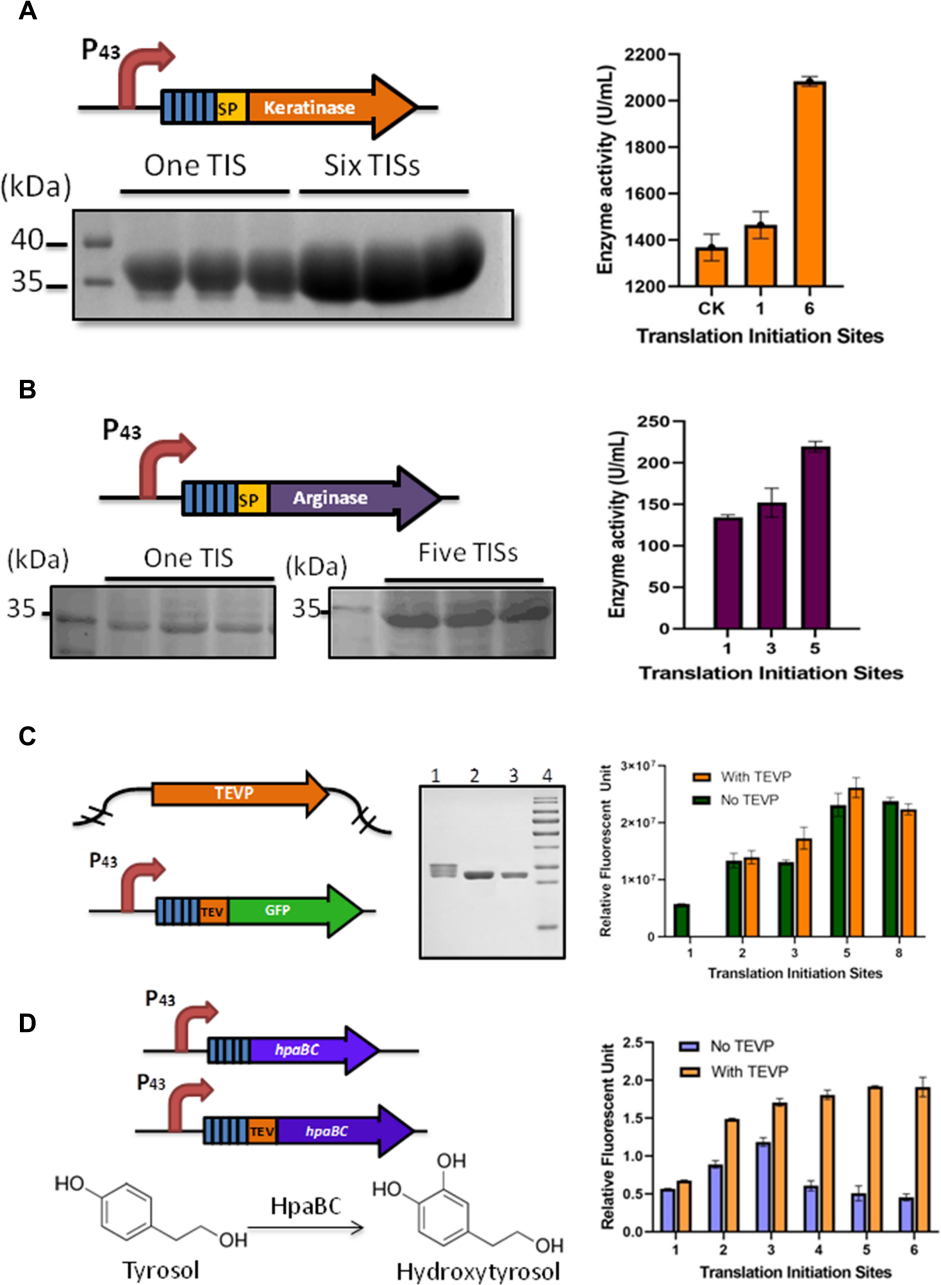
Extracellular and intracellular protein production with more than one RBS in *B. licheniformis*. (**A**) Extracellular production of keratinase via the Sec pathway with one or six RBSs. The quantity of keratinase was estimated by SDS–PAGE of concentrated medium supernatant, and the enzymatic activity was measured by the azocasein method. CK, the original P43 promoter. (**B**) Extracellular production of arginase via the Tat pathway with one or five RBSs. The quantity of arginase was estimated by SDS–PAGE of concentrated medium supernatant, and the enzymatic activity was measured by detecting the formation of l-ornithine. (**C**) Removal of the N-terminal sequences of GFP encoded by the multiple RBSs. The purified GFP was detected by SDS–PAGE. Lane 1, purified GFP with five RBSs; lane 2, purified GFP with five RBSs treated with TEV-P *in vivo*; lane 3, purified GFP with one RBS; lane 4, the ladder. (**D**) The whole-cell catalysis for hydroxytyrosol production with multipe RBSs with or without treatment with TEV-P. The experiments were performed in triplicate. The data are presented as the average numbers and the error bars are standard deviations.

*Bacillus licheniformis* is a prodigious extracellular producer of several industrial enzymes, such as proteases and α-amylases (27). There are two major transport systems for secretion of proteins in *Bacillus* species, namely the Sec pathway and the Tat pathway. We produced two enzymes to explore the application of multiple RBSs for extracellular protein production. We used the enzyme keratinase from *Bacillus amyloliquefaciens* which is secreted via the Sec pathway. We also produced the native cytoplasmic enzyme arginase which is secreted through the Tat pathway. Keratinase can hydrolyze highly rigid, cross-linked structural keratin, and has a wide range of industrial uses. Transcription of the keratinase gene with either a single RBS or six RBSs was initiated from a P43 promoter and fused with the Sec signal peptide SacCsp (Figure 3A). Compared with the keratinase with one RBS, the extracellular keratinase produced from a construct with six RBSs was 2.2-fold higher and with a commensurate 1.4-fold increase in keratinase activity (Figure 3A). Arginase is a manganese-dependent enzyme catalyzing the hydrolysis of l-arginine into l-ornithine and urea, which could be used to treat arginine-dependent cancers in mammals (28–30). The native arginase gene *rocF* was fused with the Tat signal peptide PhoDsp at the N-terminus, driven by the P43 promoter with one, three or five RBSs (Figure 3B). The amount of secreted arginase with five TISs was 2.4-fold higher compared with the arginase produced from the construct with one TIS. Enzymatic activity also increased with increasing numbers of TISs (Figure 3B). The secretion capacity might be a limiting factor for the further increase of extracellular production of keratinase and arginase. Since the N-terminal sequences encoded by multiple RBSs could be cleaved by signal peptidases along with the signal peptides during the secretion process, the extracellular proteins generated from more than one RBS had the same sizes as those with a single RBS (Figure 3A, B).

For intracellular protein production, we suspected that the N-terminal sequences encoded by the multiple RBSs might interfere with the correct folding of the target proteins. Therefore, we integrated the codon-optimized TEV protease (TEV-P) gene into the genome of *B. licheniformis*, and inserted the TEV-P recognition site between the multiple RBSs and the target genes (Figure 3C, D). After TEV-P cleavage, the number of bands associated with the different species of purified GFP produced from constructs with five RBSs decreased from five to one, sharing the same size as that with a single RBS, indicating that the N-terminal sequence was successfully removed (Figure 3C). Moreover, the TEV-P cleavage had little influence on the fluorescent intensities of GFPs (Figure 3C). Hydroxytyrosol is a powerful antioxidant that confers cell protection (31), produced from tyrosol catalyzed by two-component flavin-dependent monooxygenase HpaBC of *E. coli* (32). In order to test the feasibility of using multiple RBSs for intracellular enzyme production, we expressed *hpaBC* genes in *B. licheniformis* driven by the P43 promoter with 1–6 RBSs. When the TEV-P was absent, the production of hydroxytyrosol increased with from one to three RBSs, and then dropped dramatically for constructs with more than three RBSs (Figure 3D). These results confirmed our hypothesis that the extra N-terminal sequences encoded by multiple RBSs could undermine the function of the target enzymes. With the removal of the N-terminal sequences by TEV-P, the production of hydroxytyrosol increased gradually from constructs carrying one to six RBSs (Figure 3D), and the conversion rate achieved 86% with six RBSs.

### Multiple RBSs are capable of increasing protein output in other industrial strains

**Figure 4.**
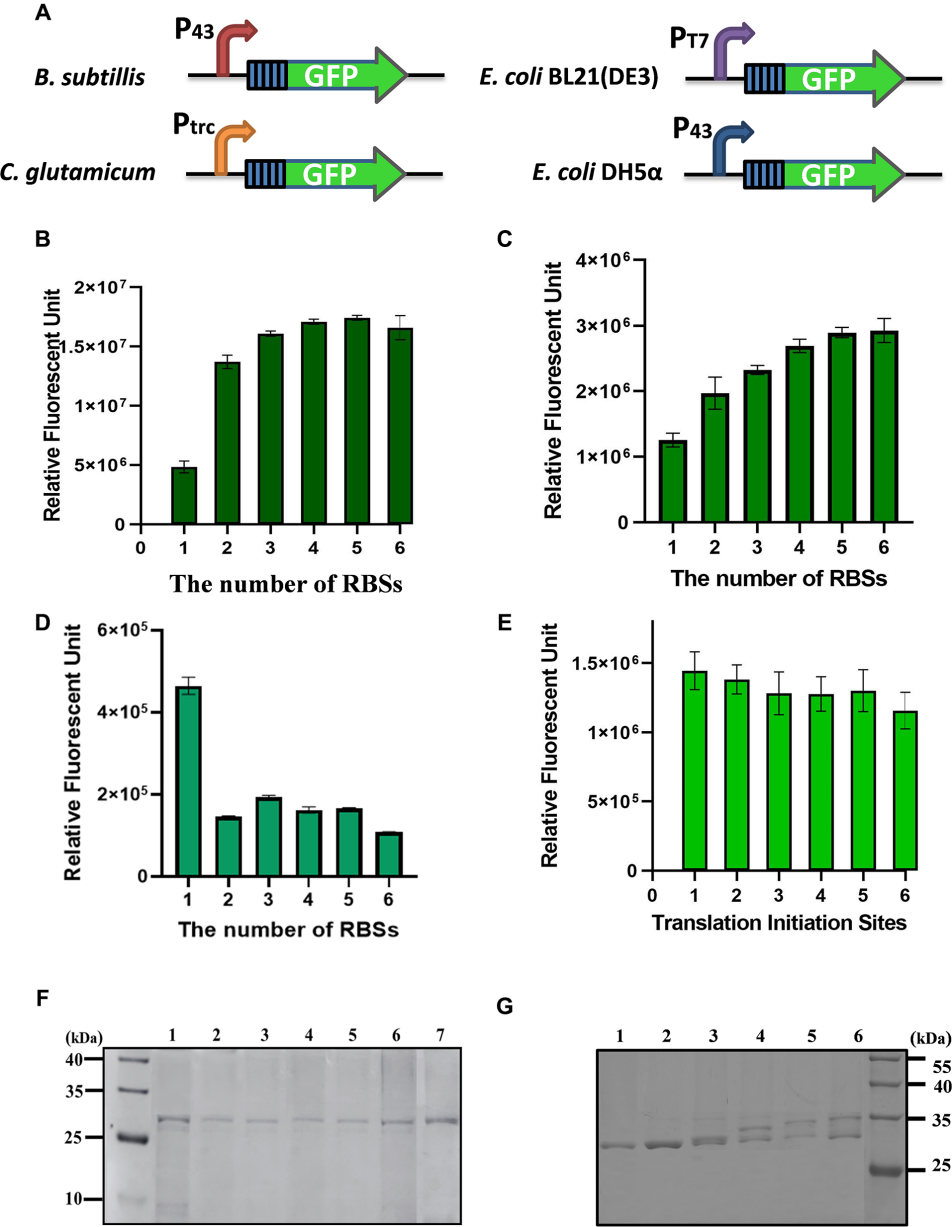
The adaptability of multiple RBSs in different industrial bacteria. (**A**) The construction of multiple RBS–GFP expression vectors in different strains. The influence of multiple RBSs on GFP expression in (**B**) *B. subtilis* 168, (**C**) *C. glutamicum* ATCC13032, (**D**) *E. coli* DH5α and (**E**) *E. coli* BL21(DE3). (**F**) Purified GFP proteins from *E. coli* DH5α expressed with 1–6 RBSs. Lane 1, CK, purified GFP proteins from *B. licheniformis* expressed with one RBS. Lanes 2–7, purified GFP proteins from *E. coli* DH5α with 1–6 RBSs. (**G**) Purified GFP proteins from *E. coli* BL21(DE3) expressed with 1–6 RBSs. Lanes 1–6, purified GFP proteins from *E. coli* BL21(DE3) with 1–6 RBSs. The experiments were performed in triplicate. The data are presented as the average numbers and the error bars are standard deviations.

The ability of more than one RBS to improve protein outputs was investigated in some other important industrial bacterial strains, including *B. subtilis* 168, *C. glutamicum* 13032, *E. coli* DH5α and *E. coli* BL21(DE3) (Figure 4). Similar to *B. licheniformis*, the multiple RBSs could effectively enhance the fluorescence intensities in Gram-positive strains *B. subtilis* and *C. glutamicum* (Figure 4B, C). However, more than one RBS caused the fluorescence intensities to drop significantly in *E. coli* DH5α (Figure 4D) and decline slightly in *E. coli* BL21(DE3) (Figure 4E). In order to identify how translation is initiated in *E. coli* with more than one RBS, we purified GFP from both *E. coli* DH5α and *E. coli* BL21(DE3). Only one protein band was observed in the SDS–PAGE gel for the purified GFP despite the number of RBSs (Figure 4F), and the size of the band is consistent with the one RBS GFP from *B. licheniformis*, indicating that the RBS closest to the GFP gene was the only active TIS at the multiple RBS sequence for *E. coli* DH5α. There were two distinct bands in the SDS–PAGE gel for the purified GFP from *E. coli* BL21(DE3) with more than two RBSs, and the sizes of the bands were consistent with the smallest and largest molecular weight of the GFP with multiple RBSs (Figure 4G), which suggested the RBSs closest to and farthest from the GFP genes were the active TISs.

It is well known that transcription and translation are tightly coupled in *E. coli* (33). Recently, researchers found that RNA polymerases outpace pioneering ribosomes in *B. subtilis* due to a faster elongation rate compared with *E. coli* (34). Similarly, the elongation rate of T7 RNA polymerase is ∼5-fold faster than for *E. coli* RNA polymerase (35), thus we suppose that transcription and translation are also uncoupled when the T7 promoter is used in *E. coli* BL21(DE3). Therefore, the tight coupling of transcription and translation might be responsible for the only TIS for the multiple RBSs in *E. coli* DH5α. However, the detailed mechanism needs further investigation. Since the coupling is not usually observed in Gram-positive bacteria (33), we speculate that the multiple RBSs could effectively enhance protein expression in other Gram-positive industrial species, such as *Lactobacillus* and *Actinomycetes*, but would probably be ineffective in Gram-negative species.

## DISCUSSION

Recently, analysis of data for 2458 bacterial genomes revealed that for all of the genes examined, ∼77.0% use an SD RBS to recruit the 30S ribosomal subunit for selection of the correct TIS on the mRNA (36). The situation where a gene possesses multiple RBSs has rarely been reported. Although a myriad of analyses have been carried out to elucidate the mechanism of prokaryotic translation initiation, most of these studies had been centered on the model organism *E. coli*. This study demonstrated that multiple RBSs located in the leader region of mRNA could lead to multiple TISs in several Gram-positive strains but only one TIS in *E. coli*, which may provide further insight into the differences in the translation initiation mechanism among prokaryotes.

Within the emerging field of synthetic biology, one of the essential challenges hampering the construction of biological systems is the lack of robust protein expression elements (37). For example, a RBS element that initiates strong translation for one gene might initiate weak expression or might not function at all for another gene. The TEs of RBSs are highly dependent on the secondary structures formed with the downstream coding sequences, causing inconsistent protein outputs (38). With the bicistronic design (BCD) (39), the translating ribosome initiated from the upstream cistron could melt the secondary structures formed at the downstream cistron by helicase activity (40), minimizing the influence of secondary structures within the RBS region on gene expression. With a similar mechanism to BCD, the multiple RBSs could also provide reliable expression for different genes. Our data confirmed that different architecture of the leader mRNA had a minor influence on protein output with the multiple RBSs (Supplementary Figure S4). Moreover, the expression levels of different genes shared a similar trend driven by the multiple RBSs (Figure 3). Therefore, the multiple RBSs could add a series of standardized genetic parts for stable protein production.

In our previous study, the optimized RBS sequence significantly enhanced the expression levels of GFP ∼50 times, compared with the original P43 promoter (13). In this study, the GFP expression with six RBSs was further increased up to five times and took up >50% of the total intracellular protein, making the multiple RBSs one of the strongest translational genetic components reported for *B. licheniformis*. The TE of six RBSs was ∼100 times higher than that of the previously constructed strong promoter coupled with the native 5′-UTR of PylB and the core promoter of PbacA (41). The protein output could potentially be further improved by coupling with promoters of higher TE. We also developed a neat method for the construction of repeat sequences in a one-step reaction (Supplementary Figure S1), making the multiple RBSs a portable element. To sum up, we developed a novel genetic component that provides efficient, robust and reliable protein expression in several industrially relevant Gram-positive bacteria for various applications.

## DATA AVAILABILITY

The data underlying this article are available in the article and in its online supplementary data.

## Supplementary Material

gkac1039_Supplemental_Files
