# Supplementary material for: Engineered multiple translation initiation sites: a novel tool to enhance protein production in Bacillus licheniformis and other industrially relevant bacteria
Source: Nucleic Acids Res. 2022 Nov 16;50(20):11979–90. doi: 10.1093/nar/gkac1039 (PMC9723656; doi:10.1093/nar/gkac1039)
Supplement: gkac1039_Supplemental_Files [file gkac1039_supplemental_files.zip › Supplementary materials0927.pdf]

Supplementary materials

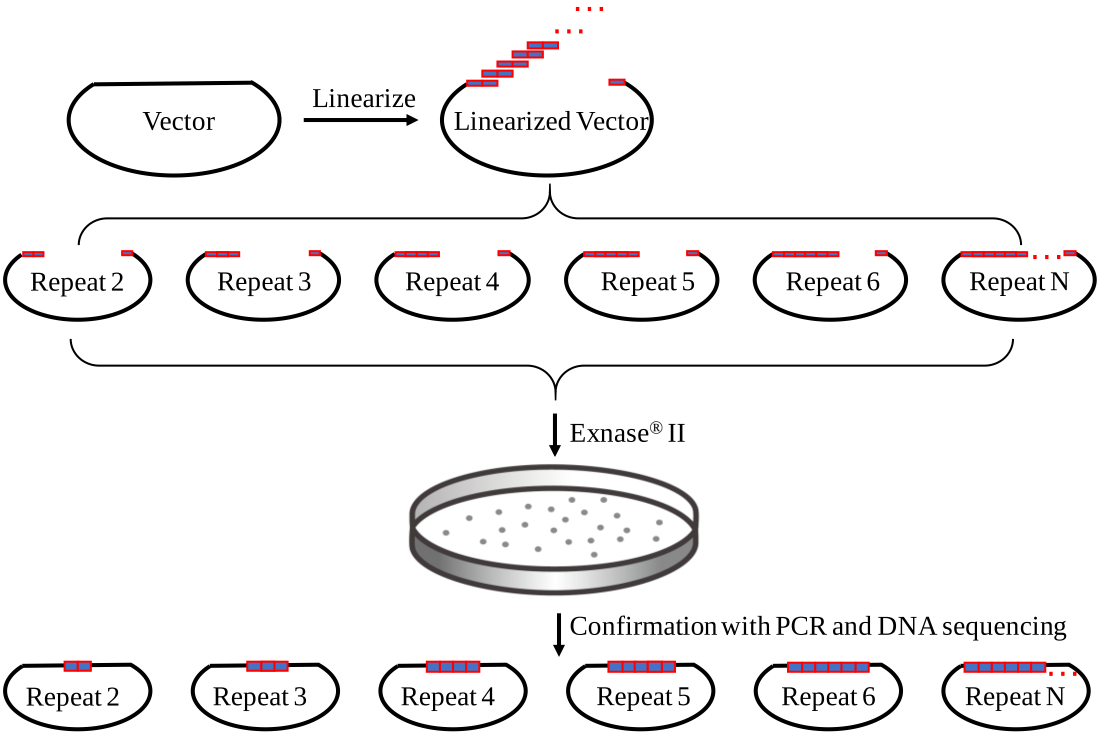

Fig.S1 The flow chart of one-step construction for the tandem repeat sequences. The primer is a sequence with double-copy repeats, and it can be bridged end to end and extended continuously, resulting in a linear plasmid carrying different numbers of repeats. The linear vector obtained by PCR was recovered, purified, and circularized in vitro using Exnase II. The circularized vector was then transformed into *E. coli* DH5α.

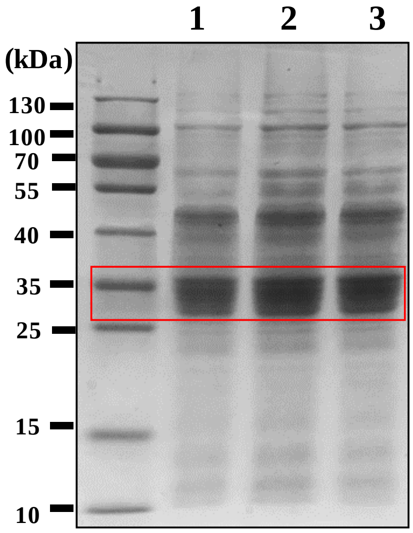

Fig.S2 The SDS-PAGE of the total intracellular protein in *B. licheniformis* harboring the GFP gene with six RBSs. The bands of GFP were highlighted by the red box. The experiments were performed in triplicate.

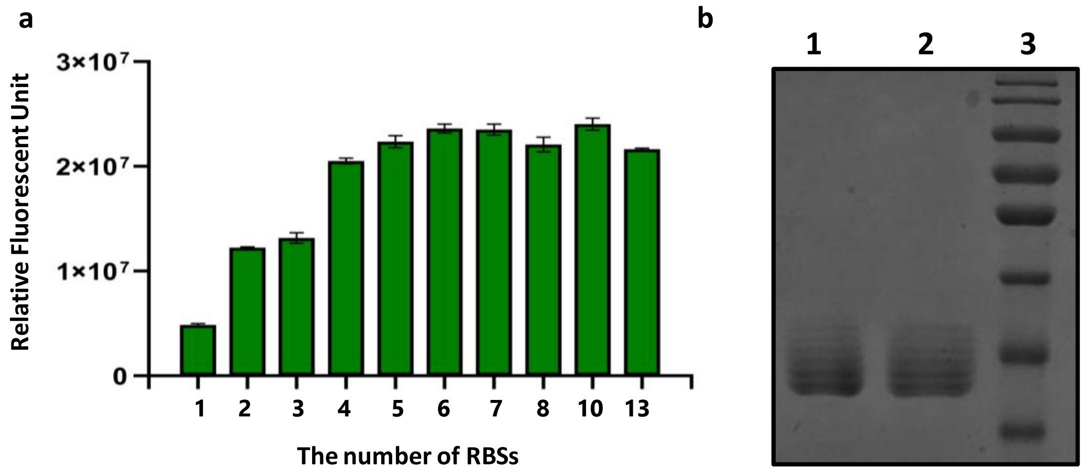

Figure S3. The influence of the number of RBSs on protein output. **a.** The fluorescent intensity of GFP with different number of RBSs. The experiments were performed in triplicate. The data were presented as the average numbers and the error bars are standard deviations. **b.** The purified GFP with seven and eight RBSs. Lane 1: GFP with eight RBSs; Lane 2: GFP with seven RBSs; Lane 3: The ladder.

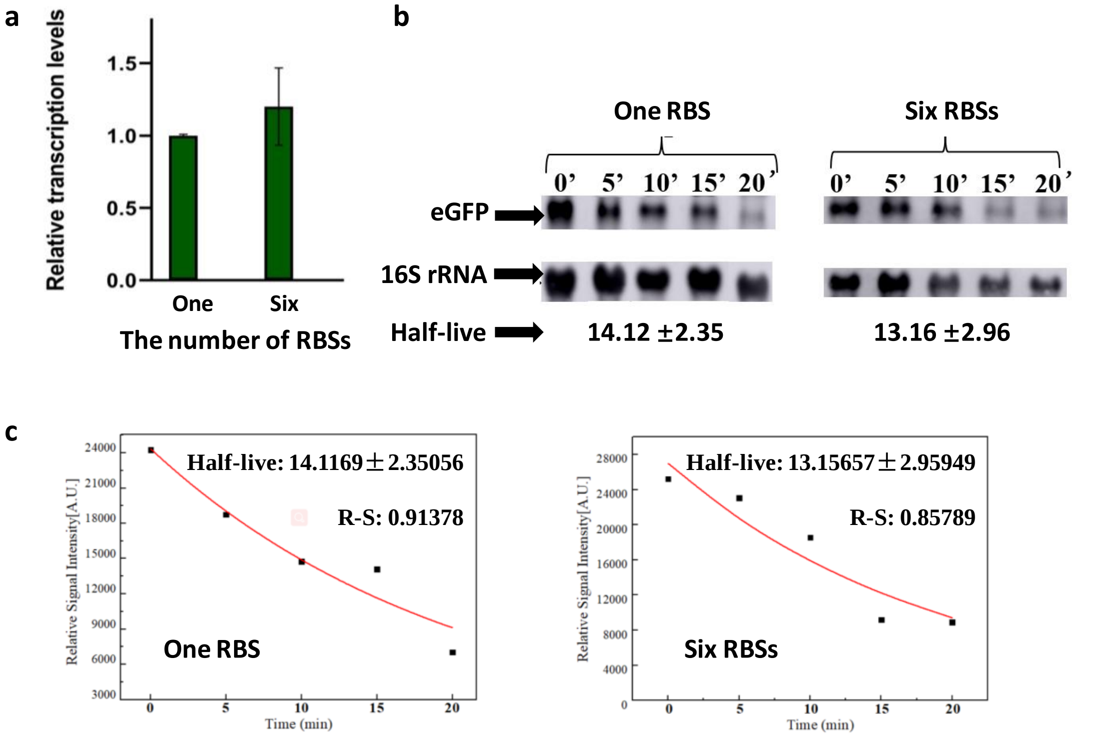

Figure S4. Transcription levels and mRNA stability play little role in translational yield. **a.** Relative transcription levels analyzed by QRT-PCR. The cells were grown to a cell density of OD_{600} 2.5 in the mid-exponential phase. 16S RNA was used as an internal control. The experiments were performed in triplicate. The data were presented as the average numbers and the error bars are standard deviations. **b.** mRNA stability analyzed by Northern blot analysis. Rifampicin (200

μg/mL) was added to the cell culture. Incubation was continued for 2 min, and 2 mL of culture samples were withdrawn for total RNA extraction at time points of 0, 5, 10, 15, and 20 min. 16S RNA was used as an internal control. **c.** The fitting curves of Northern blots for mRNA half-live determination.

a

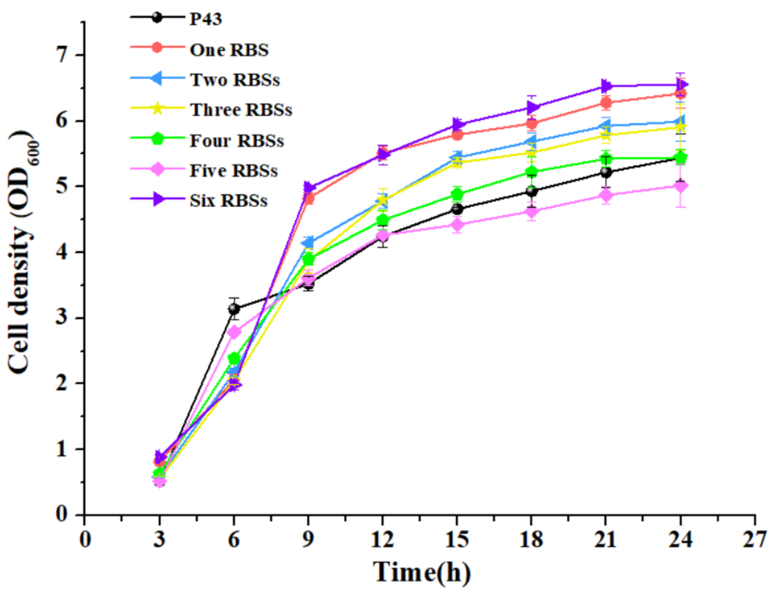

b

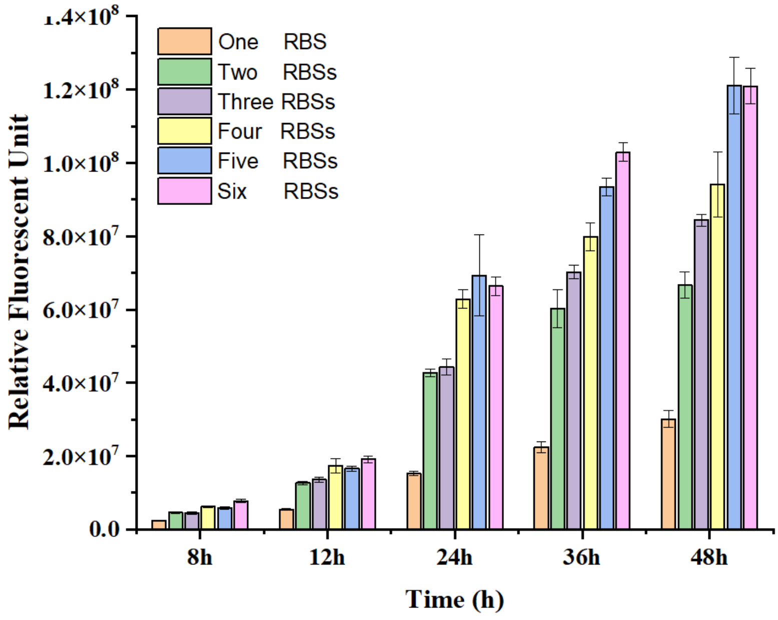

Figure S5. The growth curves and GFP expression at different growth stages of *B. licheniformis* harboring the GFP genes with different number of RBSs. **a.** The growth curves; **b.** GFP expression at different growth stages. The experiments were performed in triplicate. The data were presented as the average numbers and the error bars are standard deviations.

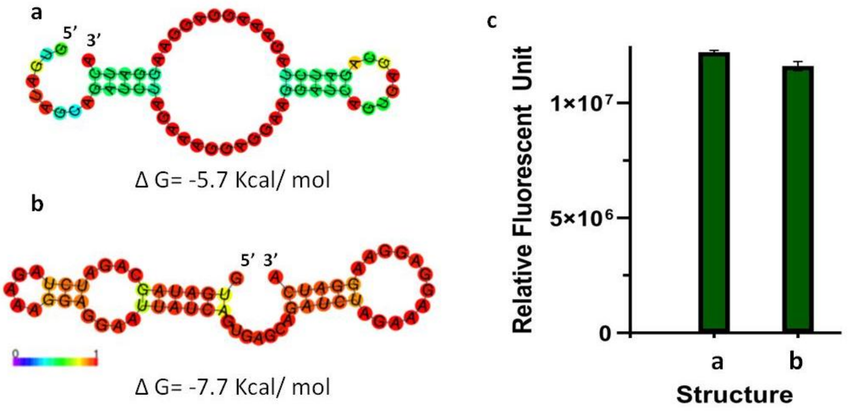

Figure S6. The influence of different structures on protein expression. **a.** The predicted secondary structure of the original two RBSs with two exposed SD sequences. **b.** The predicted secondary structure of the altered two RBSs with one SD sequence buried. **c.** The difference of secondary structures had no significant effect on protein output. The experiments were performed in triplicate. The data were presented as the average numbers and the error bars are standard deviations.

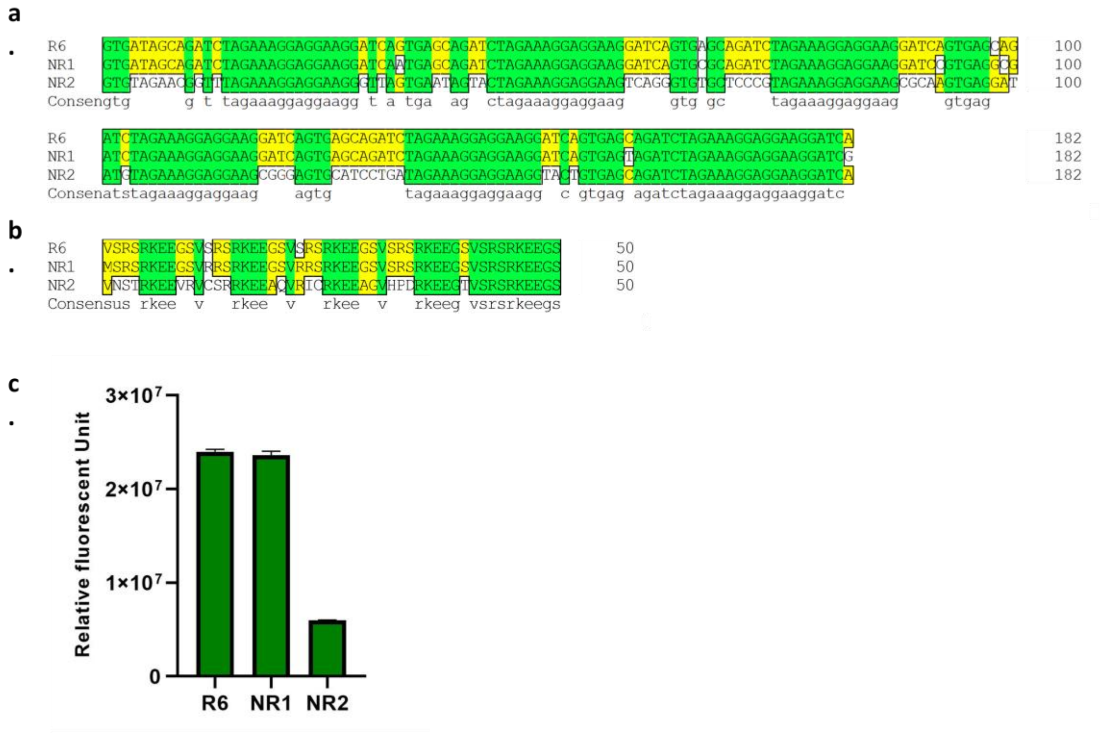

Figure S7. The influence of the different nucleic acid and amino acid sequences on protein output. **a.** The nucleic acid alignment of the original repeat sequence with two non-repetitive sequences. R6, the original six-RBS sequence; NR1, the non-repetitive sequence with changed DNA sequence through codon substitution. NR2, the non-repetitive sequence with changed amino acid sequence. **b.** The amino acid alignment of R6, NR1 and NR2. **c.** The protein outputs of R6, NR1 and NR2. The experiments were performed in triplicate. The data were presented as the average numbers and the error bars are standard deviations.

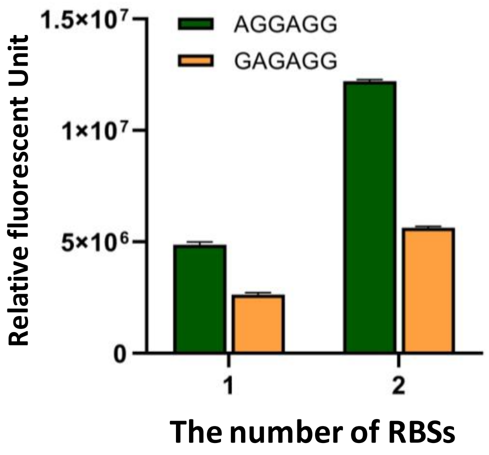

Figure S8. The SD sequence complementarity to the ribosomes affected the protein output of the plural RBSs. AGGAGG, the SD sequence completely complimentary to 3'-end of the 16S rRNA; GAGAGG, the SD sequence partially complimentary to 3'-end of the 16S rRNA. The experiments were performed in triplicate. The data were presented as the average numbers and the error bars are standard deviations.

Table S1 List of bacteria used in this study

No.	Strain name	Description	Source
1	*E. coli* DH5α	supE44Δ*lacU*169 (f80 *lacZ*ΔM15) *hsd* R17 *recA1* *gyrA*96 *thi1 relA1*	Lab storage
2	*B. licheniformis* DW2	Wild type	Lab storage
3	DW2/pHY300	DW2 harboring plasmid pHY-300	This study
4	DW2/pHY300-GFP	DW2 harboring plasmid pHY300-GFP	This study
5	DW2/pHY-2RBS-GFP	DW2 harboring plasmid pHY-2RBS-GFP	This study
6	DW2/pHY-3RBS-GFP	DW2 harboring plasmid pHY-3RBS-GFP	This study
7	DW2/ pHY-4RBS-GFP	DW2 harboring plasmid pHY- 4RBS-GFP	This study
8	DW2/ pHY-5RBS-GFP	DW2 harboring plasmid pHY-5RBS-GFP	This study
9	DW2/ pHY-6RBS-GFP	DW2 harboring plasmid pHY- 6RBS-GFP	This study
10	DW2/pHY-8RBS-GFP	DW2 harboring plasmid pHY- 8RBS-GFP	This study
11	DW2/pHY-10RBS-GFP	DW2 harboring plasmid pHY- 10RBS-GFP	This study
12	DW2/pHY-13RBS-GFP	DW2 harboring plasmid pHY- 13RBS-GFP	This study
15	DW2/pHY-RBS_{S6}-GFP	DW2 harboring plasmid pHY- RBS_{S6}-GFP	This study
16	DW2/pHY-$2RBS_{S6}$-GFP	DW2 harboring plasmid pHY-$2RBS_{S6}$-GFP	This study
17	DW2/ pHY-$3RBS_{S6}$-GFP	DW2 harboring plasmid pHY-$3RBS_{S6}$-GFP	This study
18	DW2/pHY-$4RBS_{S6}$-GFP	DW2 harboring plasmid pHY-$4RBS_{S6}$-GFP	This study
19	DW2/ pHY-$5RBS_{S6}$-GFP	DW2 harboring plasmid pHY-$5RBS_{S6}$-GFP	This study
20	DW2/ pHY-$6RBS_{S6}$-GFP	DW2 harboring plasmid pHY-$2RBS_{S6}$-GFP	This study
21	DW2/pHY-RBS_{S11} -GFP	DW2 harboring plasmid pHY-RBS_{S11} -GFP	This study
22	DW2/pHY-$2RBS_{S11}$-GFP	DW2 harboring plasmid pHY-$2RBS_{S11}$-GFP	This study
23	DW2/ pHY-$3RBS_{S11}$-GFP	DW2 harboring plasmid pHY-$3RBS_{S11}$-GFP	This study

24	DW2/ pHY-4RBS$_{S11}$-GFP	DW2 harboring plasmid pHY-4RBS$_{S11}$-GFP	This study
25	DW2/ pHY-5RBS$_{S11}$-GFP	DW2 harboring plasmid pHY-5RBS$_{S11}$-GFP	This study
26	DW2/ pHY-6RBS$_{S11}$-GFP	DW2 harboring plasmid pHY-6RBS$_{S11}$-GFP	This study
27	DW2/pHY-2RBS$_{D12}$- GFP	DW2 harboring plasmid pHY-2RBS$_{D12}$- GFP	This study
28	DW2/ pHY-3RBS$_{D12}$- GFP	DW2 harboring plasmid pHY-3RBS$_{D12}$- GFP	This study
29	DW2/ pHY-4RBS$_{D12}$- GFP	DW2 harboring plasmid pHY-4RBS$_{D12}$- GFP	This study
30	DW2/ pHY-5RBS$_{D12}$- GFP	DW2 harboring plasmid pHY-5RBS$_{D12}$- GFP	This study
31	DW2/ pHY-6RBS$_{D12}$- GFP	DW2 harboring plasmid pHY-6RBS$_{D12}$- GFP	This study
32	DW2/ pHY-2RBS$_{D15}$-GFP	DW2 harboring plasmid pHY-2RBS$_{D15}$-GFP	This study
33	DW2/ pHY-3RBS$_{D15}$-GFP	DW2 harboring plasmid pHY-3RBS$_{D15}$-GFP	This study
34	DW2/ pHY-4RBS$_{D15}$-GFP	DW2 harboring plasmid pHY-4RBS$_{D15}$-GFP	This study
35	DW2/ pHY-5RBS$_{D15}$-GFP	DW2 harboring plasmid pHY-5RBS$_{D15}$-GFP	This study
36	DW2/ pHY-6RBS$_{D15}$-GFP	DW2 harboring plasmid pHY-6RBS$_{D15}$-GFP	This study
37	DW2/ pHY-2RBS$_{D21}$-GFP	DW2 harboring plasmid pHY-2RBS$_{D21}$-GFP	This study
38	DW2/ pHY-3RBS$_{D21}$-GFP	DW2 harboring plasmid pHY-3RBS$_{D21}$-GFP	This study
39	DW2/ pHY-4RBS$_{D21}$-GFP	DW2 harboring plasmid pHY-4RBS$_{D21}$-GFP	This study
40	DW2/ pHY-5RBS$_{D21}$-GFP	DW2 harboring plasmid pHY-5RBS$_{D21}$-GFP	This study
41	DW2/ pHY-6RBS$_{D21}$-GFP	DW2 harboring plasmid pHY-6RBS$_{D21}$-GFP	This study
42	DW2/ pHY-2RBS$_{D24}$-GFP	DW2 harboring plasmid pHY-2RBS$_{D24}$-GFP	This study
43	DW2/ pHY-3RBS$_{D24}$-GFP	DW2 harboring plasmid pHY-3RBS$_{D24}$-GFP	This study
44	DW2/ pHY-4RBS$_{D24}$-GFP	DW2 harboring plasmid pHY-4RBS$_{D24}$-GFP	This study
45	DW2/ pHY-5RBS$_{D24}$-GFP	DW2 harboring plasmid pHY-5RBS$_{D24}$-GFP	This study
46	DW2/ pHY-6RBS$_{D24}$-GFP	DW2 harboring plasmid pHY-6RBS$_{D24}$-GFP	This study
47	DW2/pHY-2RBS$_{ATG}$-GFP	DW2 harboring plasmid pHY-2RBS$_{ATG}$-GFP	This study
48	DW2/pHY-3RBS$_{ATG}$-GFP	DW2 harboring plasmid pHY-3RBS$_{ATG}$-GFP	This study
49	DW2/ pHY-4RBS$_{ATG}$-GFP	DW2 harboring plasmid pHY-4RBS$_{ATG}$-GFP	This study
50	DW2/ pHY-5RBS$_{ATG}$-GFP	DW2 harboring plasmid pHY-5RBS$_{ATG}$-GFP	This study
51	DW2/ pHY-6RBS$_{ATG}$-GFP	DW2 harboring plasmid pHY-6RBS$_{ATG}$-GFP	This study
52	DW2/ pHY-2RBS$_{NC}$-GFP	DW2 harboring plasmid pHY-2RBS$_{NC}$-GFP	This study
53	DW2/ pHY-3RBS$_{NC}$-GFP	DW2 harboring plasmid pHY-3RBS$_{NC}$-GFP	This study
54	DW2/ pHY-4RBS$_{NC}$-GFP	DW2 harboring plasmid pHY-4RBS$_{NC}$-GFP	This study
56	DW2/ pHY-5RBS$_{NC}$-GFP	DW2 harboring plasmid p pHY-5RBS$_{NC}$-GFP	This study
57	DW2/ pHY-6RBS$_{NC}$-GFP	DW2 harboring plasmid pHY-6RBS$_{NC}$-GFP	This study
58	DW2/ pHY-NR1-GFP	DW2 harboring plasmid pHY-NR1-GFP	This study
59	DW2/ pHY-NR2-GFP	DW2 harboring plasmid pHY-NR2-GFP	This study
60	DW2/pHY-2RBS$_{b}$-GFP	DW2 harboring plasmid pHY-2RBS$_{b}$-GFP	This study
61	DW2/ pHY-SD2-GFP	DW2 harboring plasmid pHY-SD2-GFP	This study
62	DW2/ pHY-RBS-ker-GFP	DW2 harboring plasmid pHY-RBS-ker-GFP	This study
63	DW2/ pHY-6RBS-ker-GFP	DW2 harboring plasmid pHY-6RBS-ker-GFP	This study
64	DW2/ pHY-P43-ker-GFP	DW2 harboring plasmid pHY-P43-ker-GFP	This study
65	DW2/ pHY-RBS-Arginase	DW2 harboring plasmid pHY-RBS-Arginase	This study
66	DW2/ pHY-3RBS-Arginase	DW2 harboring plasmid pHY-3RBS-Arginase	This study
67	DW2/ pHY-5RBS-Arginase	DW2 harboring plasmid pHY-5RBS-Arginase	This study
68	DW2 /pHY-RBS-TEV-GFP-His	DW2 harboring plasmid pHY-RBS-TEV-GFP-His	This study

69	DW2 /pHY-2RBS-TEV-GFP-His	DW2 harboring plasmid pHY-2RBS-TEV-GFP-His	This study
70	DW2/pHY-3RBS-TEV-GFP-His	DW2 harboring plasmid pHY-3RBS-TEV-GFP-His	This study
71	DW2/pHY-5RBS-TEV-GFP-His	DW2 harboring plasmid pHY-5RBS-TEV-GFP-His	This study
72	DW2/pHY-8RBS-TEV-GFP-His	DW2 harboring plasmid pHY-8RBS-TEV-GFP-His	This study
73	DW2::TEVp/pHY-RBS-TEV-GFP-His	DW2::TEVp harboring plasmid pHY-RBS-TEV-GFP-His	This study
74	DW2::TEVp/pHY-2RBS-TEV-GFP-His	DW2::TEVp harboring plasmid pHY-2RBS-TEV-GFP-His	This study
75	DW2::TEVp/pHY-3RBS-TEV-GFP-His	DW2::TEVp harboring plasmid pHY-3RBS-TEV-GFP-His	This study
76	DW2::TEVp/pHY-5RBS-TEV-GFP-His	DW2::TEVp harboring plasmid pHY-5RBS-TEV-GFP-His	This study
77	DW2::TEVp/pHY-8RBS-TEV-GFP-His	DW2::TEVp harboring plasmid pHY-8RBS-TEV-GFP-His	This study
78	DW2::TEVp/ pHY-RBS-TEV-HpaBC	DW2::TEVp harboring plasmid pHY-RBS-TEV-HpaBC	This study
79	DW2::TEVp/pHY-2RBS-TEV-HpaBC	DW2::TEVp harboring plasmid pHY-2RBS -TEV-HpaBC	This study
80	DW2::TEVp/pHY-3RBS-TEV-HpaBC	DW2::TEVp harboring plasmid pHY-3RBS -TEV-HpaBC	This study
81	DW2::TEVp/pHY-5RBS-TEV-HpaBC	DW2::TEVp harboring plasmid pHY-5RBS -TEV-HpaBC	This study
82	DW2::TEVp/pHY-6RBS-TEV-HpaBC	DW2 harboring plasmid pHY-6RBS -TEV-HpaBC	This study
83	DW2/pHY-RBS-TEV-HpaBC	DW2 harboring plasmid pHY-RBS-TEV-HpaBC	This study
84	DW2/pHY-2RBS-TEV-HpaBC	DW2 harboring plasmid pHY-2RBS -TEV-HpaBC	This study
85	DW2/pHY-3RBS-TEV-HpaBC	DW2 harboring plasmid pHY-3RBS -TEV-HpaBC	This study
86	DW2/pHY-5RBS-TEV-HpaBC	DW2 harboring plasmid pHY-5RBS -TEV-HpaBC	This study
87	DW2/pHY-6RBS-TEV-HpaBC	DW2 harboring plasmid pHY-8RBS 6RBS -TEV-HpaBC	This study
88	Cog/pEC-RBS-GFP	Cog harboring plasmid pEC-RBS-GFP	This study
89	Cog/pEC-2RBS-GFP	Cog harboring plasmid pEC-2RBS-GFP	This study
90	Cog/pEC-3RBS-GFP	Cog harboring plasmid pEC-3RBS-GFP	This study
91	Cog/pEC-4RBS-GFP	Cog harboring plasmid pEC-4RBS-GFP	This study
92	Cog/pEC-5RBS-GFP	Cog harboring plasmid pEC-5RBS-GFP	This study
93	Cog/pEC-6RBS-GFP	Cog harboring plasmid pEC-6RBS-GFP	This study
94	168/pHY-RBS-GFP	168 harboring plasmid pHY-RBS-GFP	This study
95	168/pHY-2RBS-GFP	168 harboring plasmid pHY-2RBS-GFP	This study
96	168/pHY-3RBS-GFP	168 harboring plasmid pHY-3RBS-GFP	This study
97	168/pHY-4RBS-GFP	168 harboring plasmid pHY- 4RBS-GFP	This study
98	168/pHY-5RBS-GFP	168 harboring plasmid pHY-5RBS-GFP	This study
99	168/pHY-6RBS-GFP	168 harboring plasmid pHY- 6RBS-GFP	This study
100	BL21/pET-P_{T7}-GFP	*E. coli* BL21 harboring plasmid pET-P_{T7}-GFP	This study
101	BL21/ pET-P_{T7}-2RBS-GFP	*E. coli* BL21 harboring plasmid pET-P_{T7}-2RBS-GFP	This study
102	BL21/ pET-P_{T7}-3RBS-GFP	*E. coli* BL21 harboring plasmid pET-P_{T7}-3RBS-GFP	This study
103	BL21/ pET-P_{T7}-4RBS-GFP	*E. coli* BL21 harboring plasmid pET-P_{T7}-4RBS-GFP	This study
104	BL21/ pET-P_{T7}-5RBS-GFP	*E. coli* BL21 harboring plasmid pET-P_{T7}-6RBS-GFP	This study
105	BL21/ pET-P_{T7}-6RBS-GFP	*E. coli* BL21 harboring plasmid pET-P_{T7}-6RBS-GFP	This study
106	BL21/ pET-P_{T7}-6RBS-GFP-his	*E. coli* BL21 harboring plasmid pET-P_{T7}-6RBS-GFP-his	This study
107	DW2/pHY300-GFP-his	DW2 harboring plasmid pHY300-GFP-his	This study
108	DW2/pHY-2RBS-GFP-his	DW2 harboring plasmid pHY-2RBS-GFP-his	This study

109	DW2/pHY-3RBS-GFP-his	DW2 harboring plasmid pHY-3RBS-GFP-his	This study
110	DW2/ pHY-4RBS-GFP-his	DW2 harboring plasmid pHY- 4RBS-GFP-his	This study
111	DW2/ pHY-5RBS-GFP-his	DW2 harboring plasmid pHY-5RBS-GFP-his	This study
112	DW2/ pHY-6RBS-GFP-his	DW2 harboring plasmid pHY- 6RBS-GFP-his	This study

Table S2 List of plasmids used in this study

No.	Plasmid Name	Description	Source
1	pHY300PLK	*E.coli–Bacillus* shuttle vector; Amp^r in *E. coli*,Tc^r in both *E. coli* and *B. subtilis*	Lab storage
2	pHY300-GFP	pHY300PLK carrying P43-RBS-GFP-TamyL	This study
3	pHY-2RBS-GFP	pHY300PLK carrying P43-2 RBSs-GFP-TamyL	This study
4	pHY-3RBS-GFP	pHY300PLK carrying P43-3 RBSs-GFP-TamyL	This study
5	pHY-4RBS-GFP	pHY300PLK carrying P43-4 RBSs-GFP-TamyL	This study
6	pHY-5RBS-GFP	pHY300PLK carrying P43-5 RBSs-GFP-TamyL	This study
7	pHY-6RBS-GFP	pHY300PLK carrying P43-6 RBSs-GFP-TamyL	This study
8	pHY300-*kerK*	pHY300PLK carrying P43-RBS- *kerK* –TamyL, the gene *kerK* encoding keratinase	This study
9	pHY-repeat6- kerK	pHY300PLK carrying P43-6 RBSs-*kerK* –TamyL	This study
10	pHY-RBS_{S6}-GFP	pHY300PLK carrying P43-RBS_{S6}-GFP-TamyL, RBS_{S6} , the RBS with the spacer of 6 nt between SD and start codon	This study
11	pHY- 2RBS_{S6} -GFP	pHY300PLK carrying P43-2 RBS_{S6}s-GFP-TamyL	This study
12	pHY-3RBS_{S6}-GFP	pHY300PLK carrying P43-3 RBS_{S6}s-GFP-TamyL	This study
13	pHY-4RBS_{S6}-GFP	pHY300PLK carrying P43-4 RBS_{S6}s-GFP-TamyL	This study
14	pHY-5RBS_{S6}-GFP	pHY300PLK carrying P43-5 RBS_{S6}s-GFP-TamyL	This study
15	pHY-6RBS_{S6}-GFP	pHY300PLK carrying P43-6 RBS_{S6}s-GFP-TamyL	This study
16	pHY-RBS_{S11}-GFP	pHY300PLK carrying P43-RBS_{S11}-GFP-TamyL, RBS_{S6} , the RBS with the spacer of 11 nt between SD and start codon	This study
17	pHY-2RBS_{S11}-GFP	pHY300PLK carrying P43-2 RBS_{S11}s-GFP-TamyL	This study
18	pHY-3RBS_{S11}-GFP	pHY300PLK carrying P43-3 RBS_{S11}s-GFP-TamyL	This study
19	pHY-4RBS_{S11}-GFP	pHY300PLK carrying P43-4 RBS_{S11}s-GFP-TamyL	This study
20	pHY-5RBS_{S11}-GFP	pHY300PLK carrying P43-5 RBS_{S11}s-GFP-TamyL	This study
21	pHY-6RBS_{S11}-GFP	pHY300PLK carrying P43-6 RBS_{S11}s-GFP-TamyL	This study
22	pHY-2RBS_{D12}-GFP	pHY300PLK carrying P43-2 RBS_{D12}s-GFP-TamyL, RBS_{D12} , the Distance between two SD is 12 nt	This study
23	pHY-3RBS_{D12}-GFP	pHY300PLK carrying P43-3 RBS_{D12}s-GFP-TamyL	This study
24	pHY-4RBS_{D12}-GFP	pHY300PLK carrying P43-4 RBS_{D12}s-GFP-TamyL	This study
25	pHY-5RBS_{D12}-GFP	pHY300PLK carrying P43-5 RBS_{D12}s-GFP-TamyL	This study
26	pHY-6RBS_{D12}-GFP	pHY300PLK carrying P43-6 RBS_{D12}s-GFP-TamyL	This study
27	pHY-2RBS_{D15}-GFP	pHY300PLK carrying P43-2 RBS_{D15}s-GFP-TamyL, RBS_{D15} , the distance between two SD is 15 nt	This study
28	pHY-3RBS_{D15}-GFP	pHY300PLK carrying P43-3 RBS_{D15}s-GFP-TamyL	This study
29	pHY-4RBS_{D15}-GFP	pHY300PLK carrying P43-4 RBS_{D15}s-GFP-TamyL	This study
30	pHY-5RBS_{D15}-GFP	pHY300PLK carrying P43-5 RBS_{D15}s-GFP-TamyL	This study

31	pHY-6RBS$_{D15}$-GFP	pHY300PLK carrying P43-6 RBS$_{D15}$s-GFP-TamyL	This study
32	pHY-2RBS$_{D21}$-GFP	pHY300PLK carrying P43-2 RBS$_{D21}$s-GFP-TamyL, RBS$_{D21}$, the distance between two SD is 21 nt	This study
33	pHY-3RBS$_{D21}$-GFP	pHY300PLK carrying P43-3 RBS$_{D21}$s-GFP-TamyL	This study
34	pHY-4RBS$_{D21}$-GFP	pHY300PLK carrying P43-4 RBS$_{D21}$s-GFP-TamyL	This study
35	pHY-5RBS$_{D21}$-GFP	pHY300PLK carrying P43-5 RBS$_{D21}$s-GFP-TamyL	This study
36	pHY-6RBS$_{D21}$-GFP	pHY300PLK carrying P43-6 RBS$_{D21}$s-GFP-TamyL	This study
37	pHY-2RBS$_{D24}$-GFP	pHY300PLK carrying P43-2 RBS$_{D24}$s-GFP-TamyL, RBS$_{D24}$, the distance between two SD is 24 nt	This study
38	pHY-3RBS$_{D24}$-GFP	pHY300PLK carrying P43-3 RBS$_{D24}$s-GFP-TamyL	This study
39	pHY-4RBS$_{D24}$-GFP	pHY300PLK carrying P43-4 RBS$_{D24}$s-GFP-TamyL	This study
40	pHY-5RBS$_{D24}$-GFP	pHY300PLK carrying P43-5 RBS$_{D24}$s-GFP-TamyL	This study
41	pHY-6RBS$_{D24}$-GFP	pHY300PLK carrying P43-6 RBS$_{D24}$s-GFP-TamyL	This study
42	pHY-2RBS$_{ATG}$-GFP	pHY300PLK carrying P43-2 RBS$_{ATG}$s-GFP-TamyL, RBS$_{ATG,}$ the start codon GTG in the RBS is replaced by ATG	This study
43	pHY-3RBS$_{ATG}$-GFP	pHY300PLK carrying P43-3 RBS$_{ATG}$s-GFP-TamyL	This study
44	pHY-4RBS$_{ATG}$-GFP	pHY300PLK carrying P43-4 RBS$_{ATG}$s-GFP-TamyL	This study
45	pHY-5RBS$_{ATG}$-GFP	pHY300PLK carrying P43-5 RBS$_{ATG}$s-GFP-TamyL	This study
46	pHY-6RBS$_{ATG}$-GFP	pHY300PLK carrying P43-6 RBS$_{ATG}$s-GFP-TamyL	This study
47	pHY-2RBS$_{NC}$-GFP	pHY300PLK carrying P43-RBS$_{NC}$RBS-GFP-TamyL, RBS$_{NC}$, the RBS without start codon	This study
48	pHY-3RBS$_{NC}$-GFP	pHY300PLK carrying P43-2 RBS$_{NC}$sRBS-GFP-TamyL	This study
49	pHY-4RBS$_{NC}$-GFP	pHY300PLK carrying P43-3 RBS$_{NC}$sRBS-GFP-TamyL	This study
50	pHY-5RBS$_{NC}$-GFP	pHY300PLK carrying P43-4 RBS$_{NC}$sRBS-GFP-TamyL	This study
51	pHY-6RBS$_{NC}$-GFP	pHY300PLK carrying P43-5 RBS$_{NC}$sRBS-GFP-TamyL	This study
52	pHY-NR1-GFP	pHY300PLK carrying P43-NR1-GFP	This study
53	pHY-NR2-GFP	pHY300PLK carrying P43-NR2-GFP	This study
54	pHY-2RBS$_{b}$-GFP	pHY300PLK carrying pHY-2RBS$_{b}$-GFP	This study
55	pHY-SD2-GFP	pHY300PLK carrying P43-SD2-GFP	This study
56	T2(2)-Ori	*Bacillus* knockout vector; Kanr	Lab storage
57	T2-::TEVp	T2(2)-Ori-*tev*(A - B -*tev*); to express *tev*	This study
58	pHY-RBS-Arginase	pHY300PLK-P43(no RBS)- RBS -Arginase- TamyL, the gene *rocF* encoding Arginase	This study
59	pHY-3RBS-Arginase	pHY300PLK-P43-3RBS -Arginase-TamyL	This study
60	pHY-5RBS-Arginase	pHY300PLK-P43- 5RBS -Arginase-TamyL	This study
61	pHY-RBS-TEV-GFP-His	pHY300PLK-P43- RBS -GFP-TamyL-His	This study
62	pHY-2RBS-TEV-GFP-His	pHY300PLK-P43- 2RBS -GFP-TamyL-His	This study
63	pHY-3RBS-TEV-GFP-His	pHY300PLK-P43- 3RBS -GFP-TamyL-His	This study
64	pHY-5RBS-TEV-GFP-His	pHY300PLK-P43- 5RBS -GFP-TamyL-His	This study
65	pHY-8RBS-TEV-GFP-His	pHY300PLK-P43- 8RBS -GFP-TamyL-His	This study
66	pHY-RBS-TEV-HpaBC	pHY300PLK-P43- RBS -HpaBC-TamyL	This study
67	pHY-2RBS-TEV-HpaBC	pHY300PLK-P43- 2RBS -HpaBC-TamyL	This study
68	pHY-3RBS-TEV-HpaBC	pHY300PLK-P43- 3RBS -HpaBC-TamyL	This study

69	pHY-5RBS-TEV-HpaBC	pHY300PLK-P43- 5RBS -HpaBC-TamyL	This study
70	pHY-6RBS-TEV-HpaBC	pHY300PLK-P43- 6RBS -HpaBC-TamyL	This study
71	pEC-RBS-GFP	pEC XK99E-Ptrc-RBS-GFP-TamyL	This study
72	pEC-2RBS-GFP	pEC XK99E-Ptrc-2RBS-GFP-TamyL	This study
73	pEC-3RBS-GFP	pEC XK99E-Ptrc-3RBS-GFP-TamyL	This study
74	pEC-4RBS-GFP	pEC XK99E-Ptrc-4RBS-GFP-TamyL	This study
75	pEC-5RBS-GFP	pEC XK99E-Ptrc-5RBS-GFP-TamyL	This study
76	pEC-6RBS-GFP	pEC XK99E-Ptrc-6RBS-GFP-TamyL	This study
77	pET-P_{T7}-RBS-GFP	pET28a-P_{T7}-GFP-RBS-TamyL	This study
78	pET-P_{T7}-2RBS-GFP	pET28a-P_{T7}-2RBS-GFP-TamyL	This study
79	pET-P_{T7}-3RBS-GFP	pET28a-P_{T7}-3RBS-GFP-TamyL	This study
80	pET-P_{T7}-4RBS-GFP	pET28a-P_{T7}-4RBS-GFP-TamyL	This study
81	pET-P_{T7}-5RBS-GFP	pET28a-P_{T7}-6RBS-GFP-TamyL	This study
82	pET-P_{T7}-6RBS-GFP	pET28a-P_{T7}-6RBS-GFP-TamyL	This study
83	pET-P_{T7}-6RBS-GFP-his	pET28a-P_{T7}-6RBS-GFP-TamyL -his	This study
84	pHY300-GFP-his	pHY300PLK carrying P43-RBS-GFP-his -TamyL	This study
85	pHY-2RBS-GFP-his	pHY300PLK carrying P43-2 RBSs-GFP-his -TamyL	This study
86	pHY-3RBS-GFP-his	pHY300PLK carrying P43-3 RBSs-GFP-his -TamyL	This study
87	pHY-4RBS-GFP-his	pHY300PLK carrying P43-4 RBSs-GFP-his -TamyL	This study
88	pHY-5RBS-GFP-his	pHY300PLK carrying P43-5 RBSs-GFP-his -TamyL	This study
89	pHY-6RBS-GFP-his	pHY300PLK carrying P43-6 RBSs-GFP-his -TamyL	This study

Table S3 Primers used in this study

Primer name	Sequence
pHY-F	GTTTATTATCCATACCCTTAC
pHY-R	CAGATTTCGTGATGCTTGTC
GFP-blot-R	TAATACGACTCACTATAGGACATATGATCTCTTTTTTC
GFP-blot-F	GAAGGCTATGTCCAAGAAA
16S-F	CTGGTCTGTAACTGACGCTGAG
16S-R	TAATACGACTCACTATAGGAACCCAACATCTCACGACACGA
ADK-RT-F	CGTCTGACAGGACGCAGAAT
ADK-RT-R	CATTGATGTGCTTCTGCCCG
GFP-RT-F	GACGTATGGCAAACTGACGC
GFP-RT-R	ACCAGCGTATCGCCTTCAAA
16s-RT-F	ACCTAACCAGAAAGCCACGG
16s-RT-R	GTTTACGGCGTGGACTACCA
RBS-F	AGAAAGGAGGAAGGATCAATGGTCAGCAAAGGCGAA
RBS-R	TGATCCTTCCTCCTTTCTAGATCTGCTCACTGATCCTTCCTCCTTTCTAGATCTGCTCAC
RBS-YF	TTTAGAAATGGGCGTGAA
RBS-YR	CAGTTCTTCGCCTTTGCT
KerK-T5-F	AGAAAGGAGGAAGGATCAATGAGAGGCAAAAAGGTA
KerK-RBS-YR	AACGCTAAAGCAAACAGCAA
GFP-T5(A1)-F	TGATCCTTCCTCCTTTCTAGATCTGCTCACTGATCCTTCCTCCTTTCTAG
RE-32bp-GFP-R	CTAGAAAGGAGGAAGGATCAATGGTCAGCAAAGGCGAAGA

SacC-TamyL-T5-R	AAGAGCAGAGAGGACGGATTTCC
GFP-S6-F	AGAAAGGAGGGGATCATTATGGTCAGCAAAGGCGAAGA
GFP-S6-R	AATGATCCCCTCCTTTCTAGATCTGCTATCACTTTATATTTTACATAATCGC
GFP-S15-F	TGATCCTTGTTCCTCCTTTCTAGATCTGCTCACTGATCCTTGTTCCTCCTTTC
GFP-S15-R	TAGAAAGGAGGAACAAGGATCAATGGTCAGCAAAGGCGAAGA
GFP-D12-F	GATCAGAAAGGAGGAAGGATCATGGTCAGCAAAGGCGAAGAACT
GFP-D12-R	TGATCCTTCCTCCTTTCTGATCACTGATCCTTCCTCCTTTCT
GFP-D15-F	TGATCCTTCCTCCTTTCTAGATCGCACTGATCCTTCCTCCTTTCTAGATC
GFP-D15-R	ATCTAGAAAGGAGGAAGGATCAATGGTCAGCAAAGGCGAAGAACTG
GFP-D21-R	TGATCCTTCCTCCTTTCTAGATCTGCAATTCACTGATCCTTCCTCCTTTCTAGATCTGC
GFP-D24-R	TGATCCTTCCTCCTTTCTAGATCTGCATATATTCACTGATCCTTCCTCCTTTCTAGATC
GFP-ATG-R	TGATCCTTCCTCCTTTCTAGATCTGCTCATTGATCCTTCCTCCTTTCTAGATCTGCTAT
GFP-NoSC-R	TGATCCTTCCTCCTTTCTAGATCTGCTAACTGATCCTTCCTCCTTTCTAGATCTGCTA
T2-sipv-R	GGTACATTCCTCCTTTCT
T2-sipv-F	AAGAGCAGAGAGGACGGA
TEVp-F	AGAAAGGAGGAATGTACCATGGGCGAAAGCCTGTTT
TEVp-R	TCCGTCCTCTCTGCTCTTTTACAGTTGCGTCGCTTC
TEVp-AF	GTTTATGCATCCCTTAACAGAGCGGCTGATGAAGGT
TEVp-AR	AAAACATACCACCTATCATAACAGGCACGCCAAAAG
TEVp-BF	GCTGGACCGTCATCATTATGCTTTGCAGCTTGTCAC
TEVp-BR	CCCCCTTCTGTTTTTGATAGTAGAAAAGATCAAAGG
TEVp-F	GAGATTATTCGTAAAGCCGAGATG
TEVp-R	CTGTCTCCCGTGTCTTTACCCG
PEC-XK99E-F	TAACGGTTCTGGCAAATA
PEC-XK99E-R	GACCGCTTCTGCGTTCTG
PEC-repeat-F	TTCTAGATCTGCTATCACCAATTCCACACATTATACGA
RBS-pian-F	GTGATAGCAGATCTAGAAA
pEC-RBS-R	CTGCAGGTCGACTCTAGATTATTTATACAGTTCATCCA
pEC-T5-R	TCTAGAGTCGACCTGCAGGC
PET-28a-T5-F	CTGCCACCGCTGAGCAATAA
PET-28a-re-F	TTCTAGATCTGCTATCACGGAATTGTTATCCGCTCACAAT
Arginase-F	AGAAAGGAGGAAGGATCAATGACACATGAAGGACCG
Arginase-R	TCCGTCCTCTCTGCTCTTTTACAGCAGCTTCTTCCC
TEV-GFP-F	ATGGAAAATCTGTATTTTCAGGGCATGGTCAGCAAAGGCGAA
hpaBC-F	AATCTGTATTTTCAGGGCATGAAACCAGAAGATTTC
hpaBC-R	TCCGTCCTCTCTGCTCTTTTAAATCGCAGCTTCCAT
TEV-repeat-R	GCCCTGAAAATACAGATTTTCCATTGATCCTTCCTCCTTTCT
TEV-repeat-F	AAGAGCAGAGAGGACGGATTTCC
N-repeat-R	TGATCCTTCCTCCTTTCTAGATCTGCTCACTGATCCTTCCTCCTTTCTAG
